# Assessment of muscle activity using electrical stimulation and mechanomyography: a systematic review

**DOI:** 10.1186/s12938-020-00840-w

**Published:** 2021-01-03

**Authors:** Raphael Uwamahoro, Kenneth Sundaraj, Indra Devi Subramaniam

**Affiliations:** 1 Fakulti Kejuruteraan Elektronik & Kejuruteraan Komputer, Universiti Teknikal Malaysia Melaka, Tunggal, Malaysia; 2 Regional Centre of Excellence in Biomedical Engineering and E-Health, University of Rwanda, PO BOX 4285, Kigali, Rwanda; 3 Pusat Bahasa & Pembangunan Insan, Universiti Teknikal Malaysia Melaka, Tunggal, Malaysia

**Keywords:** Muscle activity, Muscle mechanics, Muscle assessment, Mechanomyography, Electrical stimulation

## Abstract

This research has proved that mechanomyographic (MMG) signals can be used for evaluating muscle performance. Stimulation of the lost physiological functions of a muscle using an electrical signal has been determined crucial in clinical and experimental settings in which voluntary contraction fails in stimulating specific muscles. Previous studies have already indicated that characterizing contractile properties of muscles using MMG through neuromuscular electrical stimulation (NMES) showed excellent reliability. Thus, this review highlights the use of MMG signals on evaluating skeletal muscles under electrical stimulation. In total, 336 original articles were identified from the Scopus and SpringerLink electronic databases using search keywords for studies published between 2000 and 2020, and their eligibility for inclusion in this review has been screened using various inclusion criteria. After screening, 62 studies remained for analysis, with two additional articles from the bibliography, were categorized into the following: (1) fatigue, (2) torque, (3) force, (4) stiffness, (5) electrode development, (6) reliability of MMG and NMES approaches, and (7) validation of these techniques in clinical monitoring. This review has found that MMG through NMES provides feature factors for muscle activity assessment, highlighting standardized electromyostimulation and MMG parameters from different experimental protocols. Despite the evidence of mathematical computations in quantifying MMG along with NMES, the requirement of the processing speed, and fluctuation of MMG signals influence the technique to be prone to errors. Interestingly, although this review does not focus on machine learning, there are only few studies that have adopted it as an alternative to statistical analysis in the assessment of muscle fatigue, torque, and force. The results confirm the need for further investigation on the use of sophisticated computations of features of MMG signals from electrically stimulated muscles in muscle function assessment and assistive technology such as prosthetics control.

## Introduction

The musculoskeletal system has been determined to play an extensive role in the locomotor function of the body. Excited by the microstimulation of the central nervous system, the system acts on the skeletal joints, generating the force required to provoke dynamic motion and posture. This force provides the joint actuator with the integral summation needed for individual muscle activation [1]. To date, the electrical response from muscle contraction has been recorded using electromyography (EMG) [2], which is detected through electrodes placed on the surface of the muscle of interest. This signal is produced by the overall contribution of active muscles beneath the skin. Thus, the recorded EMG could be contaminated by active neighboring muscles. This contamination, which is often referred to as crosstalk, has been reported in muscle coordination, motion analysis, muscle task evaluation, and prosthesis control. Different muscles are synchronously activated to contribute to a single task. Thus, scientists were aware of proper methods of screening the function of unique muscle with reduced disturbances from neighboring muscles.

NMES has been identified as an alternative tool to voluntary activation in order to generate muscle contraction; it has been determined to be an important feature to selectively activate desired muscles. Nevertheless, the changes in EMG patterns over time [3, 4] and electrical interference [5] are considered deterministic factors that might affect its proper interpretations when the targeted muscle is subjected to electrical quantities. Consequently, the development of piezoelectric, microphones, and accelerometers showed appropriate detection of the mechanical signals from the surface of skeletal muscles at a low frequency, which is known as MMG signal [6] not contaminated by electrical noise. Indeed, MMG signal represents the mechanical manifestation of muscle activity [7] and further indicates the neurophysiology reflected by the mechanical counterpart to the electrical activity of unfused active motor units [5]. Attempts made in the assessment of crosstalk [8], quantification [9], and applications in assistive technology [10] support MMG signal as an alternative to EMG signal for the screening of muscle function [11], in terms of fatigue [12], muscle force [13], and its derivative (torque) [14] as well as for prosthesis control [15] and the detection of myopathies [16].

Indeed, the frequency component of MMG signals can provide essential information on the contraction features of the muscles of interest coupled with information on the muscle fiber types and composition. For example, electrical activation of the slow- and fast-twitch muscle fibers in soleus (SOL) and vastus lateralis (VL) showed lower MMG-median frequency in SOL than in VL [17]. The mean frequency (MF) of EMG signals has been determined to mirror the conduction velocity of recruited motor units (MUs). The firing rate of recruited MUs range in 10 and 40 Hz EMG spectrum [18], whereas MMG appears at lower 10 Hz [19]. EMG is often influenced by the shape of the action potential, whereas the duration of elementary MMG and the action potential converge. Using isometric muscle action, the MMG-EMG cross-spectrum showed that unlike MMG, EMG has a low sensitivity for different types of MUs and composition [18] with long-lasting (100 µs) MU actuators than MUAP (10 µs) [19]. Thus, inference behind the features of MMG is crucial for muscle activity assessment. This fact supports the combination of MMG and NMES to provoke and record the muscle contraction, respectively. As a passive form of muscle recovery, NMES, which aims to activate and improve the function of peripheral muscles through electrical intensity delivered to intact motor nerves, found use in treating individuals suffering from dyspnea or muscle weakness [20]. Hence, the muscle integrity and force generation can be further improved by NMES-induced training, which can lead to increased muscle size and fiber type transformation [21]. In particular, MMG obtained after NMES is crucial in monitoring the reduction in muscle activation during the development of fatigue [22].

Early studies have highlighted the validity and reliability of field electrode stimulation and MMG in terms of analyzing skeletal muscle function. The available techniques for assessing human skeletal muscle activity during a diverse range of applications should be comprehensively reviewed. Few review articles have identified the muscle weakness in geriatric [20], gait speed in the lower limb [23], and the degree of spinal cord injury (SCI) [24], but the use of MMG from electrical excitation of skeletal muscles has been limited to theoretical concepts [21, 22]. Hence, this review assesses published studies on the use of MMG through NMES quantities for the assessment of muscle function. Specifically, this review identifies electrically induced MMG parameters related to health conditions of the upper limb, lower limb, and other areas of the body, and further analyzes the results from different experimental protocols used for specific muscles. This study also clarifies typical measures associated with the strengths and weaknesses of MMG from NMES-based recording methods for muscle performance. Then, assessment techniques of the muscle functions are discussed, suggesting exploratory techniques to provide novel insights that could lead to further improvements.

Collectively, intriguing issues have been concisely analyzed based on their applications to further explore the relationship between the physiological mechanism underlying a muscle and the level of physical function. In particular, this review identified studies that examined fatigue and endurance during exercise and stretching, force, power, torque, and muscle stiffness**.** In many respects, several studies reported the changes in MMG parameters caused by sensor reposition. Thus, motivated other research to develop and validate NMES electrodes and MMG sensors, as detailed in this review.

Advancements have also been made to discover the origin and characteristics of MMG, further discussing four terminologies: phonomyography (PMG), vibromyography (VMG), kinemyography (KMG), and acceleromyography. Their temporal and spectral feature representations have been used in evaluating muscle fatigue, force, and torque and in clinical monitoring using polynomial regression and linear correlation analysis. These provide evidence of MMG and NMES for the assessment of skeletal muscle function to synthesize the insights for stimulating and recording approaches in terms of the protocol design, equipment maneuvers, and performance evaluation in clinical and experimental settings. Although the review did not relate to machine learning, few studies have used support vector regression (SVR), support vector machine (SVM), and artificial neural network (ANN) to enhance the reliability of electrically evoked MMG characteristics in force, torque, and fatigue assessment. It is likely that these findings are deemed useful to further understand the muscle function and restoration and could guide for further action when specific features of MMG signals through NMES programs are required for a specific experiment.

## Methods

### Data extraction

**Fig. 1:**
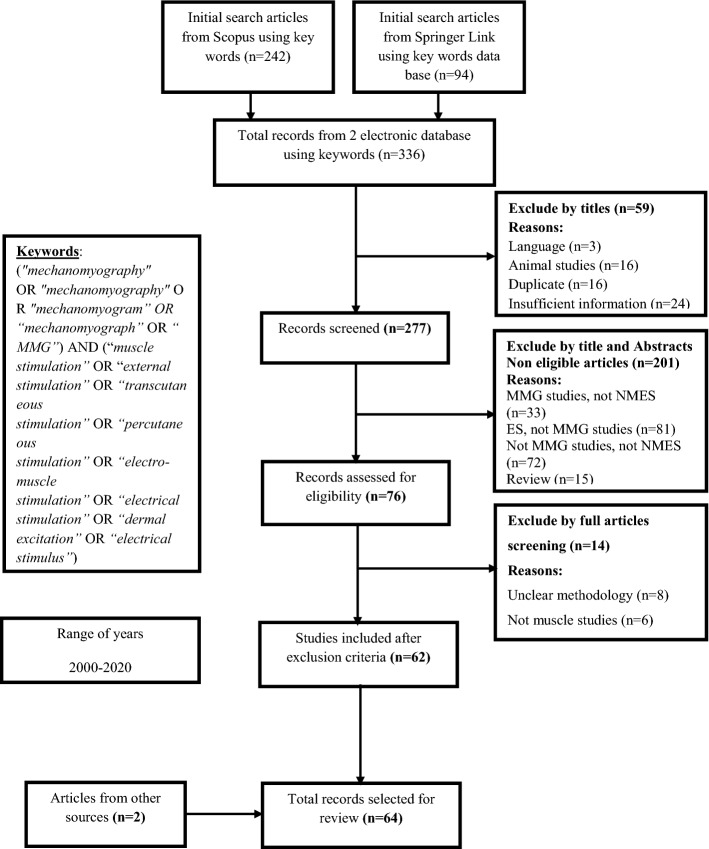
Flowchart of the article search

The studies analyzed on this research have been extracted from Scopus and SpringerLink electronic databases. These studies were identified by taking into account research works published between 2000 and 2020 using a combination of search keywords as shown in Fig. 1. This search yielded a significant number of studies that were then screened using inclusion and exclusion criteria. Initial selection evaluated the titles to identify studies on MMG through electrical muscle stimulation before full-text screening. The data extraction technique removed unreliable and nonpeer-reviewed articles and any duplicates. Studies with unclear methodology and articles written in language rather than English were removed from this review. In total, 336 articles, which were identified using this search strategy, were individually examined for eligibility for inclusion in the final study. However, 3 of the 336 articles were not included in the final screening as the manuscripts were not written in English language; in addition, 16, 16, and 24 other articles were removed from the final set as they discussed animal studies, were duplicate articles, and did not provide sufficient information. Moreover, 201 additional articles were eliminated after a review of their titles and abstracts was conducted, and this review further indicated that 33 of the studies evaluated MMG but did not include NMES, 81 discussed NMES but did not perform an MMG analysis, 72 did not evaluate MMG nor electromyostimulation, and 15 were review articles. An additional screening resulted in the exclusion of 8 studies due to an unclear methodology, and 6 other studies were further removed because they did not evaluate the muscles. The final screening resulted into 62 articles that were considered for the review, as shown in Fig. 1. Using the same eligibility criteria, screening of the bibliography in the 62 studies identified 2 articles that provided supporting information to avoid ambiguity not clarified in all the 62 studies.

### Data analysis

In total, 64 articles related to NMES and MMG were considered in this review, of which 18 highlight the application of electrically elicited MMG in fatigue assessment, 6 argued stiffness, 9 evaluated for muscle contributions to joint torque, and 4 studies addressed the assessment of force. Moreover, this investigation yielded six studies discussing the further validation of NMES technique for specific substrates in MMG signal acquisition; meanwhile, one study adopted respiratory muscle activity from the electrically elicited motor nerve. Furthermore, 13 and 6 articles, involving electrical activation of muscles, have examined the reliability of MMG parameters under experimental and clinical settings, respectively, whereas one article evaluated an approach involving MMG and electrically triggered ultrafast imaging system in real time to record small fiber contraction in the region of interest of a target muscle. NMES parameters, MMG features, type of sensors used, electrode sites, and the experimental protocol were then extracted from each article.

## Analysis and discussion of MMG and NMES

### Reliability of MMG measurements

#### Sensor sites

As shown in Table 1, four records [25–28] have been identified to examine the effects of electrode sites on MMG. The authors of one of these studies [25] found that the skinfold thickness can influence the EMG M-waves and MMG gross lateral movement recorded from electrically excited VL and rectus femoris (RF) muscles. As a measurement of the muscle belly displacement, the findings obtained in another previous study [26] revealed the efficacy of tensiomyography (TMG) in terms of detecting muscle contractile parameters with varying inter-electrode distance (IED). However, due to electrodes repositioning, a decrease in IED from ± 5 to ± 3 cm has been observed to lower the maximum muscle displacement. Thus, these researchers claimed that IED should be maintained during experiments that require electrode repositioning.

**Table 1:** Effects of electrode repositioning on the reliability of muscle contractions

Authors	Sensor and electrode type	Electrode site	Dataset	Methodology	Results and discussion
Study 1: analysis of the effect of the skinfold thickness on the MMG responses in voluntary and stimulated muscles					
[25]	MMG: active miniature accelerometer (EGAS-FS-10-/V05, Measurement Specialties, Inc., Hampton, VA, USA); force: load cell (LC402, Omegadyne, Inc., Sunbury, OH, USA)	VL and RF	17 healthy male subjects, age 21 ± 2 years, weight 81.9 ± 13.6 kg, height 1.8 ± 0.09 m; and 23 healthy female subjects, age 21 ± 2 years, weight 67.3 ± 8.9 kg, height 1.69 ± 0.07 m)	The stimulation site was determined after M-wave detected with 20 mA. The stimulation increased from 2 to 100 mA. The skinfold thickness was obtained using MMG and EMG electrodes sites and averaged to represent the thickness of each subject. MMG GLM and EMG M-waves, in terms of the log-transformation of EMG-RMS and MMG-RMS-force relationship, were correlated with the skinfold thickness	The terms and skinfold thickness were not significantly correlated, and a non-meaningful correlation was found among the skinfold thickness, EMG M-waves and MMG GLM
Remark: a significant relationship was found between the MMG GLM and the skinfold thickness of the VL and RF under non-voluntary contraction					
Future works:					
1. The influence of subcutaneous fat on MMG and EMG signals and the spectral characteristics of the test variables should be investigated					
2. The possible geometrical alteration of subcutaneous tissues and spectral features of EMG, MMG, and force during isometric contractions should be investigated					
Study 2: analysis of the reliability and effect of the inter-electrode distance on TMG parameters					
[26]	ES: self-adhesive electrodes (Compex Medical SA, Ecublens, Switzerland); TMG: (GK 40, Panoptik d.o.o., Ljubljana, Slovenia)	Right and left of the VM	18 healthy male subjects, age 22.9 ± 3.8 years, height 171 ± 10 cm, body mass 66 ± 10 kg	A current with 1-ms pulse duration and increases from 50 to 11 mA at 10-mA intervals was applied until no increase in the Dm was observed. Rater A positioned the sensors at ± 5 cm, marked ± 3 cm IED and left the room after removing electrode. Similarly, rater B performed test 2. Rater A then performed tests 3 and 4 with placements at ± 5 and ± 3 cm	Due to electrode repositioning, a decrease in the IED from ± 5 to ± 3 cm lowered the Dm (*p* < 0.01). Thus, the IED should be maintained during experiments requiring electrode repositioning
Remark: all contractile parameters showed good inter-rate reliability with the exception of Tr with an ICC of 0.99					
Future work: an experimental confirmation of TMG parameters should be conducted with different subjects, muscles groups and side-to-side asymmetry					
Study 3: analysis of the inter-changeability of TMG and ultrasound					
[27]	TMG: (TMG-BMC Ltd., Ljubljana, Slovenia)	BF, VMO and VML	10 male subjects, age 24.3 ± 2.6 years	Innervation points were detected using tetanic stimulation (0.1 ms and 10 Hz). TMG parameters were extracted after stimulation with 110 mA at 30 V. The Tc, Dm, and velocity of the radial displacement (Vr) were measured after an ultrasound B-mode scan of the same location	The contractile parameters measured with TMG and the structural parameters measured with ultrasonography were positively correlated
Remark: the interpretation of muscle contraction based on the modeled muscle shapes depends on twitch contraction					
Future work: the assessment of muscle contractile functions in terms of angle and inter excitation interval should be investigated					
Study 4: analysis of the structure and function of VMO and VML using TMG					
[28]	ES: self-adhesive anode and cathode (Axelgaard Manufacturing); TMG: (TMG-BMC, Ljubljana, Slovenia)	VML and VMO	mATPase histochemistry: 9 male subjects who experienced sudden death, age 18–44 years; TMG: 15 healthy sedentary male subjects, age 20–37 years	VML and VMO blocks frozen in nitrogen at – 196 °C, were cut into cryostat at − 20 °C for myofibrillar adenosine triphosphate activity. The muscles fibers were classified into types 1, 3a, 2b, and 2c at different pH levels TMG experiment VML and VMO received 1-kHz, monophasic pulse at 1 ms to induce Dm and three 10-s twitches followed	The TMG signal responses of electrical stimulation from two different regions has shown significant difference in ${T}_{C}$,${D}_{m}$, and ${T}_{r}$ and a constant ${T}_{d}$
Remark: the anatomical and histochemical behaviors of VMO and VML are similar, but their biological functions are different					
Future work: the determination of the biochemical function during voluntary contraction should be further considered					

In clinical settings, where muscle disuse might lead to physiological weakness, TMG can be used for estimating muscle mass recovery. Using ultrasound scans of the vastus medialis obliquus (VMO) and vastus medialis longus (VML), the thickness and pennation angle were measured, and TMG signals were then obtained at the same measurement site. The authors found that TMG was deemed reliable for the assessment of muscle degradation [27]. Besides, a previous study [28] has showed that the application of a similar twitch type to two separate regions yielded different values of contraction time (*T*_*C*_) values. Thus, TMG can be used to detect the structure and function of the VML and VMO and should be verified for other muscles.

The results from these studies on the reliability of muscle function assessment showed that the functionality of TMG to detect the small muscle displacement gave the impression to record muscle contraction at different sensors’ positions. Besides, these studies support the contention that the muscle belly displacement signal is influenced by IED. Hence, the interval between sensor positions on muscles should be kept constant in the studies of muscle activities using their mechanics. Further investigation should also shed light on the TMG characteristics considering the changes in muscle dimensions and positions for different tasks and postures.

#### Stimulation protocol and muscle responses

As presented in Table 2, nine studies [29–37] have been determined to examine the effects of the duration of the stimulation pulse on the muscle contractile properties. Some researchers [29] showed that the application of a percutaneous neuromuscular stimulation to the gastrocnemius medialis (GM) between 300 and 500 µs resulted in the displacement of the lateral belly and fixed contractile properties. The authors have recommended that the effects of skin impedance and other physiological moderators should be further examined. In another previous study [30], Yoichi Ohta evaluated the effect of nonisometric muscle contraction on joint kinematics and its reliability for detecting MMG responses. In this particular study, it was found that increases in the inter-pulse intervals or the number of stimuli yielded MMG signals that exhibited poor correlation with the changes in the excursion and angular velocity. Similarly, another study [31] revealed that the inter-day and inter-stimulus interval and the joint angle have altered the Dm, sustain time, and delay time, even though the obtained TMG parameters were deemed reliable.

**Table 2:** Effects of stimulation signal parameters on muscle contractile responses

Authors	Sensor and electrode type	Electrode site	Dataset	Methodology	Results
Study 1: analysis of the effect of changes in the duration of stimulation pulses on contractile measures					
[29]	MMG: laser sensor (model LG10A65PU: Banner Engineering, Minneapolis, MN, USA; Class 2, sensing beam with a 670-nm visible red laser, power output = 0.20 mW, beam size = 0.06 × 0.8 mm, resolution = 10 μm)	BB	10 healthy male subject, age 19–33 years	Stimulation pulses were increased from 50 to 500 $\mu s$ until no further increase in the MMG Dm was detected	The duration of the pulse impacted the muscle contractions reflected by MMG
Remark: the lateral displacement and rate of muscle contraction decreased from 50 to 300 µs, and none of the fibers were maximally activated below 300 µs					
Future work: other muscles and the effect of skin impedance on other moderators must be evaluated in the future					
Study 2: effect of non-isometric muscle activation on joint parameters and MMG					
[30]	MMG: 9-mm^2^ accelerometer (thickness = 4.5 mm, mass = 0.75 g, sensitivity = 500 mV/g where g = 9.8 m/s^2^; MP110-10-101, MediSens INS, Japan)	TA	8 healthy male subjects, age (means ± SDs) 27 ± 2.9 years, height 173 ± 9.1 cm, weight 73 ± 5.5 kg	The ankle joint and MMG were measured after one, two, three, four, seven and eight stimulation pulses separated by a 1–5-min rest; the 10-ms (100-Hz) pulses were administered at an inter-pulse interval of 10, 20, 30, 40, 50, 80 and 100 ms	At different inter-pulse intervals or numbers of stimuli, the MMG exhibited a poor correlation with the changes in joint kinematics
Remark: torque changes should not be considered for the control of the initial joint movement using functional electrical stimulation					
Study 3: analysis of the effect of the inter-pulse duration on muscle contractile parameters					
[31]	ES: stimulating electrodes (Compex Medical AS, Ecublens, Switzerland); TMG: (BMC Ltd., Ljubljana, Slovenia)	BB	13 male and 2 female subjects, age 29.5 ± 7.4 years, height 176.9 ± 9.2 cm, body mass 78.7 ± 14.9 kg	A 10-s ES was delivered to the BB positioned at 10, 45, 90° with the arm at rest for 10–20 s on 2 separate days. The delay time (Td), contraction time (Tc), sustained time (Ts), relaxation time (Tr) and maximal displacement (Dm) were compared between the 2 days	The test–retest reliability of TMG parameters was significant for 2 days
Remark: the interpretation of muscle contraction in terms of modeled muscle shapes depends on twitch contraction					
Future work: the possible maximal stimulation response should be verified					
Study 4: analysis of the effect of submaximal contraction on the MMG response					
[32]	Accelerometer (13 × 18 mm, 0.94 g; MMA7260Q, Free scale)	RF	13 healthy male, age 21.3 ± 6.5 years, mass 79.3 ± 6.08 kg, height 179 ± 10.71 cm	The femoral nerve was excited by nine NMES frequencies at 1 kHz modulated at 20, 25, 30, 35, 40, 45. 50, 75 and 100 Hz	The relationship between NMES frequencies and MMG responses is not linear
Remark: a high frequency does not impact the mechanomyographic characteristics and thus does not affect for the application of a neuroprosthetic device					
Study 5: analysis of the effect of post-activation potentiation on the MMG of synergistic muscles					
[33]	EMG: 11-mm pick-up diameter, 25 mm inter-electrode distance; MMG: uniaxial accelerometer (dimensions = 9 × 9 × 5 mm, mass = 0.75 g, model MP101-10, MediSens, Japan)	MG and SOL	8 male subjects, age (means ± SDs) 26.86 ± 3.7 years, height 176.1 ± 6 6.3 cm, mass 71.2 ± 6 6.1 kg	Before and after a 10-s MVC, a 500-µs pulse was delivered every 1 min for 5 min, and one stimulus was applied after 10 min. The evoked MMG was measured before and after 10-s supramaximal plantar flexion	The potentiation of both muscles with the plantar flexion angle was investigated. The MG showed a higher MMG amplitude than SOL at DF and NP
Remark: a 10-s dorsiflexion and neutral position of the MG showed greater potentiation than SOL, but no significant difference in PAP for plantar flexion was found between the two conditions					
Study 6: analysis of the effect of post-activation potentiation on MMG					
[34]	MMG: (MP110-10-101, MediSens, Inc., Japan; sensitivity = 500 mV/g, where g = 9.8 m/s^2^)	MG	10 healthy male subjects, age 25.8 years, height 170.3 ± 4.8 cm, weight 67.8 ± 7.5 kg	After supramaximal stimulation to determine the M-wave and a 10-min rest, three isometric contraction at a 5-s interval were delivered for each twitch stimuli. Twitch contractions were evoked 2, 15, 30, 60 and 180 s after the MVC	No change in the M-wave was found after MVC. MMG measured after the evoked twitch contractions reflect changes in muscle contraction
Remark: after PAP, the evoked MMG-PP represents the contractile properties of the muscle					
Study 7: analysis of the intensity and contraction velocities of skeletal muscles					
[37]	MMG: accelerometer (ADXL330, Analogue Devices, Inc., Norwood, MA, USA); EMG: Ag–AgCl electrodes (EL503; Biopac Systems Inc.)	Soleus muscle	3 male and 5 female subjects, age 19 ± 1 years	An H-M recruitment curve was mapped for the soleus muscle by increasing the 0.1-ms square wave at 1.0- to 5-V increments with a 10-s rest interval until an M-wave was recruited	The maximum sEMG corresponding to H-reflex and M-waves showed a moderate correlation between HM and ${MMG}_{PP}$
Remark: the time-to-maximum intensity (TTMax) was longer at a low stimulation intensity and declined with increases in the intensity					
Future work: further studies should account for the body composition, muscle fiber composition, gender and training					
Study 8: effect of unilateral surface stimulation session on the contralateral limb					
[35]	MMG: 6.5-g accelerometer (K-Beam 8305A; Kistler, Amherst, MA, USA); EMG: Ag/AgCl bipolar surface electrodes (Blue Sensor M-00-S; Medicotest, Ølstykke, Denmark)	RF	36 healthy right-footed male subjects, age 25.8 ± 1.3 years, weight 75.0 ± 2.1 kg, height 178.3 ± 1.1 cm	After stimulation at 100 Hz with a 300-µs pulse duration, a cycle of 10 s on and 10 s off was applied for relaxation of the RF of the non-dominant leg of the 18 RS group for 10 min	MMG, EMG and maximum voluntary isometric contraction from the dominant leg before and after stimulation showed no changes in the MMG activity of the RF (*p* < 0.05)
Remark: the lack of mechanical changes could be due to the short exposure time to the stimulus					
Future work: the influence of a long exposure time to the stimulus on the tension, rigidity, mass and length of the muscle should be investigated					
Study 9: analysis of the effect of the staircase phenomenon on neuromuscular blockade (NMB) monitoring					
[36]	AMMG (train of four (TOF)–Watch SX; Organon, Dublin, Ireland)	Abductor pollicis	17 males and 7 females in group C, age 45.9 years, BMI 25.6 kg/m^2^; 17 males and 5 females in group S, age 47.9 years, BMI 25.1 kg/m^2^	Group C received 2-Hz TOF every 15 s over 20 min, and rocuronium was injected into the other hand Group S was tetanically stimulated (50 Hz, 5 s, and 50 mA)	Prior to acceleromyography, tetanic stimulation resulted into reduced onset and recovery times of AMMD amplitudes
Remark: sp has no influence on the TOF ratio					
Future work: the effect of sensitivity on monitoring NMB function should be investigated					

The literature also demonstrates that the NMES frequency does not correlate with the MMG frequency response. Papcke et al. [32] reported that during NMES at 5% maximum voluntary isometric contraction, the application of a 20-, 25-, 30-, 35-, 40-, 45-, 50-, 75-, and 100-Hz stimulation frequency did not correlate with any of the three axes analyzed during the Cauchy wavelet-based frequency analysis of MMG signals from the RF. Specifically, the mechanical characteristics of the RF have exhibited a frequency of 20–25 Hz that differs from and is not governed by the stimulation frequency. Hence, this study revealed that a high frequency does not impact the MMG characteristics and, therefore, does not affect the application of MMG in neuroprosthetics.

Postactivation potentiation (PAP) has been determined to be dependent on the joint angle and fiber composition. Specifically, a previous study [33] found a difference in the inter-muscle MMG due to PAP of the SOL and GM muscles at the ankle joint. After MVC in the neutral and dorsiflexion directions, a single stimulus was delivered, and a recording uniaxial accelerometer was positioned between two distal and proximal EMG electrodes on each muscle belly. After applying a stimulation protocol similar to that applied in pre-MVC, simultaneous and individual MMG measurements yielded a higher peak-to-peak MMG amplitude from the GM than the SOL in the neutral and dorsiflexion directions. As both muscles were potentiated, it was determined that the magnitude of the PAP depended on the joint angle and composition of fiber types. Another previous study [34] used percutaneous neuromuscular electrical stimulation of the tibial nerve and twitch torque to evaluate the MMG and EMG responses of the GM. Thus, a positive correlation was found for the ${\mathrm{MMG}}_{\mathrm{pp}}$ with both pT and acceleration of twitch torque development (${\mathrm{d}}^{2}\mathrm{T}/{\mathrm{dt}}^{2}$). Moreover, it was determined that the evoked MMG signals mirror the PAP force and that the mechanical changes in the muscles are deemed related to an increase in the twitch contraction. These findings reveal the reliability of MMG in characterizing the contractile properties of a muscle after PAP. Moreover, another study [35] has noted a stable MMG response before and after electrical excitation of non-dominant RF, despite increases in the EMG response and strength. In this particular study, it was suggested that a high exposure time can improve the mechanical behaviors. Meanwhile, another study [36] has evaluated the onset time, recovery time, and rate of changes in piezoelectric myographic amplitudes after a tetanic stimulation of the ulnar nerve, wherein reductions in the onset and recovery times were observed prior to measured myograph.

Specifically, the wavelet-based intensity analysis was reported to provide insight into the spectral characterization of individual muscle contractile properties. Using both MMG and surface (sEMG), the correlation between the MMG and H-reflexes was determined to support the hypothesis that muscle mechanics may provide insight into muscle recruitment strategy. The authors suggest that future work should evaluate the clinical implication of the MMG signals corresponding to H-reflexes and M-waves [37].

Insights from the reliability of muscles recruitment and function monitoring have shown that the stimulation pulse duration [29], number of stimuli [30], pulse rate [31], and joint angles [28, 34] provide global insights for elucidating the relationship between muscle contraction and recovery of their mechanical properties. Furthermore, the muscle fiber types and joint angles have been identified as two of the factors affecting the PAP [34]. Because MMG provides useful information on the musculoskeletal system, various MMG features, such as the onset time, recovery time, and relaxation time, should be examined after PAP [36, 37]. In contrast, no correlation was found between the frequency of stimulation and the MMG response [32]. Based on this perspective, the reliability of muscle recruitment and mechanical features remains an issue that should be addressed in future studies. In addition, the tissue underlying the skin, viscoelastic properties, and motor tendon units could alter the behaviors of the MMG signal. Therefore, muscle excitation and feature extraction techniques should be verified for output repeatable measurements. Interestingly, the variable and fixed contractile properties of the BB muscle’s belly for known stimulation pulses were observed. Hence, a new parameter, namely, M + H, from the wavelet intensity analysis using both MMG and sEMG through NMES coupling encourages further verification of the technique for muscle recruitment using the stimulating signal pulses duration and frequencies of different muscles.

### Studies on the electrodes for NMES and sensors for MMG measurement

As shown in Table 3, six studies [38–43] have been determined to discuss the performance of developed electrodes and sensors in MMG and electrical muscle stimulation settings, further describing their ongoing development. In one study [38], a silver (Ag)-polydimethylsiloxane (PDMS) composite was developed and used for electrical muscular activation. After fabrication, single and array electrodes were then used to excite the BB muscle. Their functionalities have been evaluated by comparing the MMG signals detected after electromyostimulation of the BB using the newly developed single and array electrodes to the ones detected using commercial NMES electrodes. As per the results, it was shown that the MMG signals obtained with single rather than array electrodes appeared to have a higher range. As such, ES of a specific muscle by single Ag–PDMS electrode is deemed more effective than array electrodes. Thus, single electrode yields a better signal-to-noise ratio compared with array electrodes. Array electrodes, on the other hand, have been determined to be crucial for wearable devices, particularly in cases in where nonuniform contact region could be present and where continuous NMES is needed.

**Table 3:** Overview of the use of ES and MMG in sensor validation

Study 1: validation of Ag–PDMS substrate for the electrostimulation of muscles					
Authors	Sensor and electrode type	Electrode site	Dataset	Methodology	Results and discussion
[38]	Ag–PDMS composite	BB	Not reported	Single and array electrodes composed of Ag/Ag–PDMS and PDMS/OHP substrates were used, and MMG signals were recorded for testing purposes	The responses were similar to those obtained with a single commercial electrode, with an average peak of 7000–10,000 mV
Remark: the Ag–PDMS can be bent and stretched, which was a limitation of the Ag–OHP paper electrode					
Study 2: evaluation of a lever indentor and a moving magnet galvanometer for MMG recording					
[39]	ES: galvanically isolated stimulator (MYOSTIM); MMG: scanner galvanometer; Force: ankle dynamometer (RAFOLT and GALLASCH, 1996)	Calf muscles	6 healthy subjects	9 stimulation pulses were delivered to achieve a contraction of 70 N, whereas the indentor was adjusted to 0.1, 0.5 and 5 N. Under isometric contraction of the calf muscles, an ankle dynamometer was used to record the surface response from the gastrocnemius muscle	The amplitudes of MMG-RMS showed direct increases during contraction
Remark: a unity cross-correlation coefficient confirmed the validity of using a galvanometer for recording MMG signals					
Future work: further studies should verify indirect stimulation					
Study 3: analysis of the accuracy of an accelerometer for tensiomyography					
[40]	TMG: optical encoder (4 µm, 0.25 mm^−1^; a spherical tip of 12 mm^−2^); MMG: displacement accelerometer	BB	Not reported	A single-twitch stimulus consisting of a 1-ms, 20-mA square pulse was delivered to the BB using two self-adhesive electrodes. The double integration of the acceleration records were compared with the optical encoder records	The MEM accelerometer efficiently detects short-term small muscle displacement
Remark: the difference in ${D}_{m}$ recorded from an accelerometer and a displacement sensor and the time parameter must not differ by more than 0.05 mm and 0.5 ms, respectively					
Study 4: characterization of muscles and subcutaneous tissues					
[41]	ES: Ag–AgCl; DMMG: (LK-G80, Keyence, Osaka); AMM: (MP-110-10-101, MediSens, Saitama)	TA	6 healthy males, age 22–25 years	A monopolar rectangle pulse with a 500 µs in width and an inter-pulse interval of 600 ms was applied	Good identification of the longitudinal and transversal mechanics of the muscle, subcutaneous tissue and skin was achieved
Remark: the natural frequency of an acceleration sensor fluctuates more than that of a displacement sensor, but the latter is limited to longitudinal muscle mechanics					
Future work: the effect of the mass of subcutaneous tissue on the natural frequency should be investigated					
Study 5: analysis of the reliability of MMG and a laser-displacement sensor					
[42]	MMG: laser-displacement sensor (LDS; class 2 laser; model LG10A65PU, Banner Engineering Australia) and contact-displacement sensor (CDS; Positek P101 Stand Alone Linear Position Sensor)	RF	16 female and 14 male subjects, age (means ± SDs) 22 ± 2.7 years, height 1.70 ± 0.09 m, body mass 68.9 ± 11.0 kg	Stimulation with a voltage of 400 V, a pulse duration of 200 µs, and a current amplitude of 40—280 mA with 10-mA increments was applied until the muscle reached full displacement; five successive single twitches were delivered at maximum intensity	Both sensors showed good test–retest reliability over the four testing sessions
Remark: the two sensors are not interchangeable: the CDS appears to be more sensitive to muscle belly displacement, whereas the LDS shows increased sensitivity					
Future work: the allocation between the stimulation interval and ½ Tr should be well monitored in order to ensure an efficient recovery time for all MMG parameters					
Study 6: evaluation of MC sensors					
[43]	ES: 5–9 cm (RehaTrode, Hasomed GmbH, Magdeburg, Germany); MMG: (TMG-BMC Ltd., Ljubljana, Slovenia)	RF	9 SCI subjects, age 41.6 ± 14.5 years	A stimulation with 35 Hz, 200 µs and an amplitude of 70—110 mA was followed by MC recording to predict the torque	The MC sensor torque and the dynamometer knee torque were linearly correlated
Remark: MC sensor measurements are reliable and can be used as an alternative for fatigue estimation					
Future work: an experiment using a high number of subjects with SCI and different protocols should be performed to validate the use of an MC sensor for real-time data transmission					

Another previous study [39] has evaluated the applicability of a galvanometer-attached lever and skin indentor in MMG signal detection. During isometric contraction of the calf muscles, a dynamometer was used to record the surface response 3 cm from the motor point of the GM muscle. The contraction force was then recorded by a load cell. After the application of single-twitch electric intensity, an accelerometer was utilized to detect the target contraction on the skin surface. During evaluation of the effect of indention and muscle contraction, the amplitude RMS showed a proportional increase during contraction; furthermore, constant mean frequencies were observed. The accelerometer and galvanometer amplitudes were then preserved at 200 N, but their double differentiation output reportedly decreased by 9%. A unity cross-correlation coefficient confirmed the validity of using a galvanometer for recording MMG signals.

Interestingly, the availability of different forms of MMG recording devices has encouraged the examination of the validity of a micromachined acceleration sensor for TMG [40]. Simultaneous recordings, coupled with a linear optical encoder-based displacement sensor and accelerometer, were obtained to acquire the muscle belly displacement at 100 data points per 1 s; in this experiment, a bandwidth of 2300 Hz was observed after a single-twitch square-wave stimulus of the BB. Based on previous studies, the researchers found that the results agreed with the minimal allowable threshold amplitude and a window length of 1 mm. The standard deviation of the double integration of the linear displacement MMG increased over time. The mean relative error, the maximum displacement (Dm), and ½T_r_ were 0.02 mm, 0.6, and 3 ms, respectively. Therefore, this study has confirmed that the microelectromechanical system (MEMS) accelerometer can be used to detect short-term small muscle displacement.

Meanwhile, another study [41] has examined the performance of displacement and acceleration transducers using a system identification method, with electrical stimuli as the input and acceleration (AMMG) or displacement (DMMG) as the output. Based on this study, it was determined that DMMG at a natural frequency of 3 Hz was suitable as a longitudinal mechanical feature, whereas AMMG was found to be of advantage when used for reflecting both the longitudinal and transverse mechanical characteristics of muscles, subcutaneous tissue, and the skin. However, a 3-Hz higher fluctuation was noted for AMMG compared with DMMG. Although the sensors showed good performance in measuring underlying events, some evidence shows the importance of the transduction rate and sensitive parameters. In another previous study [42], it was found that a laser-displacement sensor (LDS) and a contact-displacement sensor (CDS) exhibited satisfactory reliability after four tests conducted over 2 weeks. Despite the recovery Tc and D_max_, the half-relaxation time (½T_r_) exhibited poor recovery to its pre-fatigued value during the recovery period. Despite the slower Tc of CDS, it was found that the sensor might detect individual muscle actions, whereas noncontact LDS exhibits some limitations, and this uniformity is thought to limit the inter-changeability of these sensors.

Meanwhile, another previous study [43] has compared the average and peak torques measured using a commercial dynamometer, with the torque estimated using a muscle contraction sensor (MCS) during functional electrical stimulation evoked muscle contraction. The results showed that the signals obtained using MCS and dynamometer were strongly correlated, which indicated that these sensors can be used instead. Although some limitations such as different responses of the LDS and CDS, AMMG and DMMG, effectiveness of VMG than MCS in muscle fatigue assessment for SCI population [44], there exist proof that confirms their validity in the myographic signal recording. Based on recent insights on selecting MMG sensors [45, 46], experimental verification with similar conditions and recording sites may provide better conclusions. A comparison of MMG recorded using MEMS accelerometer for TMG measurement showed that the MEMS accelerometer is reliable in terms of detecting small muscle displacement. AMMG was also found to measure both longitudinal and transverse muscle characteristics and subcutaneous tissues. In addition, the lack of fast recovery of ½Tr in LDS and slower CT response in CDS appear to be somewhat favor accelerometers as MMG sensors. Other available sensors such as piezoelectric sensors and MCS require further comparisons with MEMS accelerometers.

### Fatigue assessment

In total, 18 of the 64 records have been identified to describe the examination of muscle fatigue using MMG and NMES protocols. Among these 18 studies, 10 articles in Table 4 utilized various techniques for the quantification of fatigue, 4 articles in Table 5 documented fatigue and muscle physiology, and 4 articles in Table 6 reported the relevance of fatigue and endurance. In one study [47], the development of fatigue was examined at 10%${\mathrm{MVC}}_{10\mathrm{ min}}$ wrist extension during a 20-min pre-experiment and its recovery over 10, 30, 90, and 150 min under low-force contraction. The force analysis was conducted at 1, 20, and 100 Hz. The low-frequency fatigue (LFF) was measured as the response of the ratio of the 20- to 100-Hz of stimulating signal. The ${\mathrm{EMG}}_{\mathrm{RMS}}\mathrm{ and }{\mathrm{MMG}}_{\mathrm{RMS}}$ values were observed to increase at the force ratio of 20/100 Hz at 10% maximum MVC within 10 and 30 min of recovery. No significant changes were noted in the 80% MVC test. In addition, no change in the mean power frequency (MPF) values was observed at 5% MVC, whereas a decrease before the experiment at 10% MVC and after 90–150 min was detected. Therefore, these findings reveal that low-force muscle contraction leads to prolonged LFF, as identified by EMG and MMG. These results are supported by [48] where LFF was observed for 2 h after a 5% MVC hand grip for 30 min.

**Table 4:** Assessment of muscle fatigue using MMG and ES

Authors	Sensors and electrode type	Electrode site	Dataset and electrode sites	Methodology/electrical stimulation protocol	Results and discussion
Study 1: examination of prolonged low-force fatigue due to prolonged low- and high-frequency ES					
[47]	MMG: uniaxial accelerometer (Bang & Olufsen Technology, Denmark, diameter = 17.6 mm, weight = 2.9 g, 20 pC/ms^−2^, 0.1 to 800 Hz); EMG: bipolar Ag/AgCl (Blue Sensor N-00-S, Medicotest, Denmark, diameter = 6 mm); Force: Alpha Beam 250 N (BLH Electronics, USA)	ECR	3 healthy and 4 healthy male subjects, age 27–54 years, height 1.52–1.84 m, weight 56–92 kg, body mass index: 20.9–27.2 kg/m^2^	Arm flexed at 90º, static wrist extension for 10 min at 10% MVC; over 150 min, the subject was administered a 10-s train at 1 Hz, two 2.5-s trains at 20 Hz, and two 2-s trains at 100 Hz s, with a 30-s rest between each train The ES was adjusted to obtain 30% of the MVC at 100 Hz, each pulse lasted 0.7 s	LFF showed electromechanical efficiency in the low-force and control experiment for up to 9000 s, as reflected in MMG more than the EMG
Remark: a low-force test is recommended for fatigue development in muscle and recovery prior to low-force exertion					
Study 2: analysis of the changes in electromechanical delay components of skeletal muscle exposed to fatigue					
[49]	EMG: model ELSCH004 (OT Bioelettronica, Turin, Italy); MMG: accelerometer (model ADXL103, Analogue Devices, Norwood, MA, USA; weight < 1.0 g, sensitivity = 1000 mV/g, range = ± 1.7 g); force: load cell (model SM-1000 N, Interface, Crowthorne, UK, linear operation between 0 and 1000 N)	GM	20 healthy subjects, age (means ± standard deviations) 23.1 ± 4.2 years, body mass 74.3 ± 11.2 kg, stature 1.77 ± 0.08 m	12 blocks of 10-s stimulation at 35 Hz, pulse duration of 340 us, a duty cycle of 9 s on/1 s off for a duration of 120 s was delivered after 2-Hz, a 5-mA increasing amplitude was administered for motor unit stimulation	All of the delays lengthened the contraction based on different onsets and kinetics. The changes in the cross-bridge and muscle tendon unit (MTU) mechanical properties occurred later compared with the electrochemical events
Remark: the delay in the lengthening of mechanical events suggests that these were the most affected by fatigue					
Future work: further studies are needed to evaluate the electrochemical and electromechanical alternations at the motor tendon units and muscle junctions under fatigue conditions					
Study 3: evaluation of the inter- and intra-operator reliability of the measurements and the effects of fatigue on different ${\mathrm{Delay}}_{\mathrm{TOT}}$ components					
[50]	MMG: accelerometer (model ADXL103, Analogue Devices, Norwood, MA, USA; weight = < 1.0 g; sensitivity = 1,000 mV/g; range = ± 1.7 g); force: load cell (mod. SM-1000 N, Interface, UK); EMG: linear array of four electrodes (model ELSCH004, OT Bioelettronica, Turin, Italy)	GM	16 healthy male subjects, age (means ± SDs) 25.0 ± 3.9 years, body mass 77.5 ± 13.8 kg, stature 1.79 ± 0.08 m	A biphasic 50-Hz pulse with a 340-µs duration at 110% of the M-wave amplitude was administered during a 3-s tetanic stimulation with a 5-min rest before a fatiguing tetanic pulse of 120 s at 35 Hz was delivered to the GM. The pre-fatiguing pulse was repeated at 1, 2, and 7 min	The ICC was 0.874–0.996, and the SEM was 0.78 and 6.61% before fatigue. The reliability was 0.781 to 0.981, and the SEM was 1.78 to 8.71%. All the variables were reliable within an inter-parameter operability of 0.847 to 0.999 after fatigue
Remark: the intra- and inter-operator reliability of individual ${Delay}_{TOT}$ components increases, and the results provide a valid indication for the monitoring of physiological and pathological changes					
Study 4: effects of fatigue on ${\mathrm{Delay}}_{\mathrm{TOT}}$ components and their inter-session and inter-day reliability					
[51]	MMG: accelerometer (model ADXL103, Analogue Devices, Norwood, MA, USA; device weight = < 1.0 g; sensitivity = 1000 mV/g; range = ± 1.7 g); EMG (model ELSCH004, OT Bioelettronica, Turin, Italy)	GM	17 healthy male subjects, age 24.3 ± 3.4 years, body mass 77.8 ± 14.3 kg, stature 1.79 ± 0.08 m	A biphasic 50-Hz pulse with a 340-µs duration at 110% of the M-wave amplitude was administered during a 3-s duration tetanic stimulation with a 10-min rest before a fatiguing tetanic pulse of 120 s was delivered at 35 Hz to the GM	${\mathbf{D}\mathbf{e}\mathbf{l}\mathbf{a}\mathbf{y}}_{\mathbf{T}\mathbf{O}\mathbf{T}}$, R-∆t F-MMG_*p*-*p*_, and ${\mathbf{D}\mathbf{T}}_{\mathbf{S}\mathbf{l}\mathbf{o}\mathbf{w}}$ were positively correlated. Fatigue changed the duration of the experiment and the start of force decays but had no effect on its duration
Remarks:					
1. Fatigue might prolong R-EMD components, which might indicate physiological recovery after physical or rehabilitation exercises					
2. After fatigue, the constant elongation of the R-∆t MMGR-F without changes in R-∆t F-MMGPP might be due to increases in the spatial relationship between blood vessels and muscle fibers					
Future work: the reoccupation of the squeezed out interstitial fluid might be attributed to alterations in the return of the MMG signal to baseline. Further studies are needed					
Study 5: evaluation of the reliability of MMG for determining the evoked changes during muscle relaxation					
[52]	MMG: (model 10ADXL103, Analogue Devices, Norwood, MA, USA; weight = < 1.0 g; 1000 mV/g; range = ± 1.7 g); EMG: (model ELSCH004, OT Bioelettronica, Turin, Italy); Force: (model SM-1000 N, Crowthorne, UK)	GM	23 HV male subjects, age 25.1 ± 3.8 years, body mass 78.2 ± 15.3 kg, stature 1.81 ± 0.05 m	A biphasic 50-Hz pulse for 340 µs at 110% of the M-wave amplitude was administered during a 3-s tetanic stimulation with a 10-min rest before a fatiguing tetanic pulse for 120 s at 35 Hz was delivered to the GM	High ICC values between the MMG and force parameters was obtained at different experimental sessions on different days
Remark: the correlation between ${\mathrm{R}-\mathrm{MMG}}_{\mathrm{PP}}$ and force confirm the effect of mechanisms of muscle fatigue that modify the extent of velocity and force relaxation					
Future work: full relaxation of the muscle under voluntary dynamic and isometric contractions should be investigated					
Study 6: analysis of the use of TMG and PMT to evaluate peripheral fatigue-induced alterations in mechanical and contractile properties					
[53]	ES: (5 cm^2^, Axelgaard, USA); Contractile properties detection: TMG (BMC Ltd., Ljubljana, Slovenia)	GM	21 HV male subjects, age (means ± SDs) 21.3 ± 3.4 years, height 182.0 ± 6.1 cm, mass 79.5 ± 10.0 kg	1-ms pulse; amplitude of 20 mA, 10-mA increase to evoke Dm. An inter-pulse rest of 10 s was used to lower both fatigue and potentiation. The fatigue protocol was 15 pulses (1 every 100 ms) at approximately 110 mA over 5 min	PMT from the plantar flexor increased, and the TMG Dm decreased, which confirmed muscle stiffness after fatigue
Remark: a decrease in the TMG Dm is not associated with Vc, but the results are limited to healthy individuals and cannot be applied to non-superficial muscles					
Future work: future verification of fatigue changes in various cohorts should be performed					
Study 7: prediction of the muscle fatigue in SCI patients using SVM					
[54]	ES: RehaTrode (HASOMED, Germany); MMG: accelerometer (Sonostics BPS-IIVMG transducers; 20–200 Hz, 30 V/g, 10 g)	VL, RF, and VM	5 SCI patients classified as Class A and B according to ASIAIS	30-min NMES-cycling at 120 mA, 30 Hz, biphasic, pulse width of 400 ± 400 μs. The MFCC and RMS MMGs were trained and tested based on SVM	Contractions correctly identified as non-fatigued and fatigued had higher MFCC compared with RMS values
Remark: both MFCC and RMS showed that fatigued muscle contractions overlapped with non-fatigued muscle contractions, which resulted in insufficient prediction					
Future works:					
1. Multiple experimental trials and analyses of the effects of the window size of MMG signals should be performed to improve the accuracy of the SVM classifier					
2. The nature of MMG signals that influence physiological properties and the physical environment should be investigated					
Study 8: assessment of fatigue based on MMG and torque responses					
[55]	ES: two 9-cm × 15-cm self-adhesive electrode (RehaTrode, HASOMED, Germany); MMG: (Sonostics VMG BPS II Transducer, frequency = 20–200 Hz, sensitivity = 50 V/g)	VL, VM and RF	5 male and 1 female subjects with complete chronic SCI	30 Hz, 400-μs pulse width, 90–120 mA to the quadriceps; 30 Hz, 60–120 mA, 300 μs was used to elicit NMES-leg cycling exercise from the quadriceps; and 58–90 mA was applied to the hamstrings for 30 min	The MMGmpf, MMGrms and NMES-cycle time altered similarly to the epT during pre- and post- fatigue in a 2-day test
Remark: the high fatigability of RF might lead to limited cycling exercises in SCI subjects					
Future work: the reliability of muscle fatigue assessment during functional recovery using NMES-cycling in SCI patients should be further investigated					
Study 9: quantification of fatigue under repeated functional electrical excitation					
[56]	MMG measurement device: LIS311, STMicroelectronics, USA	TA	21 healthy male subjects, age 24.0 ± 2.0 years, height 174.1 ± 6.9 cm, weight 75.3 ± 13.2 kg	60 Hz, 240 μs, relaxation times of 0.5 s, 2.0 s, 0.5 s, and 1.0 s. MMG signals were collected for 30 min. During MVC and ES, the torque was measured offline using a dynamometer	During muscle fatigue, the MMGpp, convex-hull volume, and convex-hull area linearly decreased with decrease in the mean and median frequencies
Remark: the Lempel–Ziv symbolization technique exhibited the best performance for complex MMG feature reduction					
Future works:					
1. Based on Lempel–Ziv symbolization, further studies should address the required threshold for individuals under NMES					
2. Repeated evoked fatigue was observed under isometric conditions, and isokinetic and/or isotonic conditions should be investigated					
Study 10: analysis of the mechanical fatiguing phenomenon that develops during ES in sport training or rehabilitation protocols					
[57]	**MMG:** ADXL202JE (Analogue Devices, Inc., Norwood, MA, USA); **EMG**: silver bar (diameter = 1 mm, length = 5 mm, inter-electrode = 10 mm); **force**: load cell (Interface, model SM-100 N, operation range = 0 and 100 N)	BB and VL	10 healthy subjects, age 20–50 years	6 potentiation pulses of 100 Hz with a rest of 1 s followed by a fatigue protocol of 50 Hz for 2 s and 2 Hz for 25 s. Normalized MMG and pT were linearly used for fatigue evaluation	The torque and MMG decreased linearly from 100% of their initial values to 50 and 60% for the BB and to 43 and 47% for the VL, respectively
Remark: accurate screening of muscle mechanical fatigue should eradicate muscle tendon unit insertion and joints

**Table 5:** Studies of fatigue and muscle physiology

Authors	Sensor and electrode type	Electrode site		Dataset	Methodology	Results and discussion
Study 11: relationship between temporal and spectral MMG features during fatiguing electrical muscle excitation						
[58]	MMG: freescale MMA7260QMEM tri-axial accelerometer, sensitivity = 800 mV/G at 1.5 g	RF and VL	10 HV subjects, age 28.30 ± 6.58 years 10 SCIV subjects, age 32.06 ± 9.68 years		A single pulse at 1 kHz based on a 15% duty cycle was delivered with a rest of 2 to 5 min, and the maximum electrical stimulation was determined based on the voltage required to vary the knee angle from approximately 90° to 40°	Both the HV and SCI analyses yielded correlation coefficients of − 12 up to − 0.82
Remark: the negative correlation between ${\mathrm{MMG}}_{\mathrm{RMS}}$ and ${\mathrm{MMG}}_{\mathrm{MF}}$ justify their divergence due to fatigue and motor neuron adaptation						
Future work: strategies for differentiating the timing among muscle fibers events during the NMES-based recovery process should be investigated						
Study 12: analysis of electrical and mechanical behaviors of stimulated pre-fatigued muscles						
[59]	EMG: silver bars (diameter = 1 mm, length = 10 mm, inter-electrode = 10 mm); MMG: accelerometer (model ADXL202JE, Analogue Devices, Norwood, MA, USA); force: load cell (model #SM-200 N, Interface, UK; operation range = 0 and 200 N)	GM	11 healthy male subjects, age 21 ± 2 years, body mass 75 ± 4 kg, stature 1.79 ± 0.06 m		A set of three 50-Hz, 10–100-mA, 307-µs pulses lasting 5 s with a 1-min rest between contractions was delivered before and after the fatiguing protocol (35 Hz for 120 s) and stretching maneuvres (elongation up to 45 s with 15-s rest periods)	EMG, MMG and force features decreased and recovered after a 420-s rest period. A stretching protocol reduced the MMG and force signals
Remark: passive stretching remains questionable during a cooldown routine						
Future work: studies on passive muscle tendon units should help verify the force reduction after stretching						
Study 13: evaluation of the features of human muscle and mechanomyography from interpolated twitch methods						
[60]	**MMG:** uniaxial accelerometer (9-mm square, thickness = 4.5 mm, mass = 0.75 g, sensitivity = 500 mV/g where g = 9.8 m/s^2^; MP110-10–101, MediSens INS, Japan)	GM	12 male subjects, age 27 ± 2 years, height 0.5 ± 5.2 cm, weight 68.5 ± 9.7 kg		The plantar flexion force was measured at 20, 40, 60, 80 and 100% followed by a supramaximal 1-ms stimulus to the twitch resting torque. The superimposed twitch amplitude, MMG amplitude and ultrasonic images at each force level were recorded	The superimposed MMG amplitude and the extent of fascicle shortening with increasing intensities showed similar patterns
Remark: superimposed MMG might strongly mirror changes in the muscle architecture rather than the twitch amplitude						
Study 14: analysis of passive stretching on the electromechanical properties of muscles						
[61]	**EMG**: silver bar electrodes (diameter = 1 mm, length = 10 mm, inter-electrode distance = 10 mm); **MMG**: one accelerometer (ADXL202JE, Analogue Devices, Norwood, MA, USA); **force:** load cell (model SM-200 N, Interface, UK; operation range = 0 and 200 N)	GM	12 healthy male subjects, age (means ± standard errors) 23 ± 1 years, body mass 76 ± 5 kg, stature 1.79 ± 0.005 m		Six electrical stimulations with a rest period of 5 s between stimulations. The force signal was induced by two impulses of 100 Hz for 307 µs during a 1-s period. The stretching signals during five maneuvres lasting 45 s with a 15-s rest were obtained	Acute passive stretching altered the mechanical but not the electrical properties
Finding: attention should be paid to the use of MMG to examine stretch-induced changes in the mechanical properties of skeletal muscles

**Table 6:** Use of MMG for studying endurance and fatigue

Authors	Sensor and electrode type		Electrode site	Dataset		Methodology	Results and discussion
Study 1: analysis of torque and MMG fluctuations at various joint angles							
[63]	Triaxal accelerometer-based MMG sensor (WAX3, Axivity, Newcastle upon Tyne, UK)		Erector spinae of the forearm and wrist flexors	5 male and 3 female subjects, age 22.9 ± 2 years, height 1.8 ± 0.1 m, weight 69.8 ± 14.9 kg		Pulses of 25 to 35 mA at pulse durations/intervals of 200/50 µs were delivered, and the muscle twitch contraction was measured. The endurance protocol consisted of 2 Hz, 4 Hz, and 6 Hz (3 min each) with 5 s of no stimulation between each stage	The EI values were reasonably reproducible, particularly those obtained with the 2- and 4-Hz stimulations
Remark: in young healthy individuals, the erector spinae muscle has a lower endurance index than the leg muscles							
Future work: the experiment was conducted on young healthy subjects; thus, the application of the system for evaluating lower back pain in the healthy population should be investigated							
Study 2: analysis of the effect of exercise on endurance							
[62]	Accelerometer-based MMG sensor		GM	56-year-old female with muscle sclerosis	A 3-min, 2-, 4- and 6-Hz stimulation signal was applied, and the endurance index was determined as the percentage of the acceleration at the last stage of the stimulation frequency relative to the peak accelerometer records		The walking endurance and oxidative capacity were improved
Future works:							
1. The effect of voluntary exercises on muscle plasticity and the role of muscle oxidative capacity on assisting individuals with MS should be evaluated							
2. The use of a large sample size for examining the role of muscle plasticity to improve the walking function in people with MS should be investigated							
Study 3: evaluation of rider time and virtual distance in SCI							
[64]	ES: two self-adhesive surface electrodes (oval, 2″ × 4″); MMG: accelerometers (Entran EGAS, weight = 1 g, 2–150 Hz, gain of 25)		VM, RF and VL	12 SCI subjects, mean age 37 years, height 1.8 m, mass 80.6 kg		An electrical stimulation of 50 Hz for 500 µs was increased to a current of 140 mA to maintain a cadence of 32 RPM over 0.4 s and applied every 1.88 s	An improved electrically activated cycling alternated the activity of synergistic muscles in SCI, and different RMS values of MMG signals were obtained from all the muscles
Remark: the MMG amplitude decreased due to muscle fatigue and the virtual distance, and both the stimulation and co-activation protocols yielded the same mechanical output							
Future work: the use of improved stimulation techniques to evaluate improvements during a longer training practice without an external rider should be evaluated							
Study 4: analysis of endurance and feelings of fatigue in FRDA							
[65]	MMG: tri-axial accelerometer (WAX-9; Axivity, UK); ES: electrodes (5.08 cm by 10.16 cm, Pro Advantage by NDC)	Forearm flexor		10 healthy subjects, 16 FRDA subjects		The forearm flexor muscles were stimulated with 3 min of ES at 2, 4, and 6 Hz with a 5-s rest between stages	A correlation was found among the mitochondrial capacity, disease severity and muscle-specific endurance
Remark: people affected by FRDA exhibit lower forearm muscle endurance than ABs							
Future work: MMG and NIRS measurement methods are correlated with disease severity, and this correlation should be further investigated							

Meanwhile, four studies [49–52] have evaluated muscle behavior after fatiguing stimulation for 120 s, followed by tetanic stimulation at 1, 2, and 7 min. One of these studies [49] evaluated the changes in electromechanical delay components from the GM. The ${\mathrm{Delay}}_{\mathrm{TOT}}$ values were determined based on the RMS and MF of the EMG, MMG, and force. All measurements were obtained from a time frame of 250 µs during fatiguing stimulation. During a 120-s stimulation, ${\mathrm{MMG}}_{\mathrm{RMS}}$ was noted to show stable patterns over the first 90 s, but then decreased until the end of the stimulation. The MF of MMG presented a strong reduction during the first 10 s; then, it monotonically decreased until the end of stimulation. In contrast, the ${\mathrm{EMG}}_{\mathrm{RMS}}$ increased by 40% during the first 70 s before it returned to its initial values, whereas the MF components presented reductions starting after the first 10 s until the end of the stimulation. In this study, various electrochemical and mechanical components were identified, and the researchers reported that the detected delay correlated with the tested EMG, MMG, and force parameters. In addition, the researchers claim that the mechanical events ($\Delta$ t MMG-F) provided a highly reliable measure of fatigue. These findings were consistent with those obtained in another study [50] that investigated the effect of fatigue on the delay in force development (${\mathrm{Delay}}_{\mathrm{TOT}}$) from GM during 35-Hz fatiguing stimulation for 120 s. Before and after the induction of fatigue, a couple of tetanic stimulations were administered, with a resting interval of 10 min, and the effect of fatigue was indicated by the reductions in ${p}^{\mathrm{F}}$ and ${\mathrm{MMG}}_{p-p}.$ The results also showed that fatigue lengthened the delay, which in turn affected its electromechanical and electrochemical components.

Similarly, another study [51] has examined the impacts of fatigue on electromechanical delay components and assessed the inter-day and inter-session reliability during relaxation after tetanic and fatiguing stimulation protocols, same as those used in the above-mentioned study [50]. The results demonstrated that the increase in ${\mathrm{R}-\mathrm{Delay}}_{\mathrm{TOT}}$ paralleled the increase noted in electrochemical components and the first mechanical component measured after fatigue, whereas the second mechanical component, which was an electromechanical component measured at the onset of the force decay and the negative MMG peak values, did not change. Therefore, a low contribution to the ${\mathrm{R}-\mathrm{Delay}}_{\mathrm{TOT}}$ was found.

The aims of another study [52] were twofold: (1) to examine the correlations between peak-to-peak MMG and other force parameters and (2) to validate the inter-day reliability of mechanical parameters of the MMG signals induced by a tetanic stimulation before and after the NMES to fatigue. The authors reported that ${p}^{\mathrm{T}}$, ${\mathrm{MMG}}_{\mathrm{PP}}$, ${\mathrm{R}-\mathrm{MMG}}_{P-P},$ and the acceleration of force development, the slope, and relaxation decreased after fatiguing stimulation, but the relaxation time (RT), CT, and τ were noted to increase. The relaxation MMG (${\mathrm{R}-\mathrm{MMG}}_{P-P}$) correlated with the ${\mathrm{MMG}}_{P-P}$, slope, τ, ${p}^{\mathrm{T}},$ and the acceleration of force development (${\mathrm{D}}^{2}\mathrm{RFD}$) before and after fatigue. Due to the high intraclass correlation coefficient between the MMG and force parameters obtained from different experimental sessions on different days, it was concluded that the MMG is a viable alternative to force in terms of examining fatigue-induced changes during muscle relaxation.

Furthermore, Macgregor et al. [53] have examined muscle fatigue based on muscle tension through the temporal and spatial displacement recorded from GM before and after supramaximal stimulation through a process known as TMG. As per the result of this study, a significant (*P* < 0.001) decline was noted in the peak force measured with MVC; thus, it was hypothesized that this decline was in line with the decrease in the TMG Dm (*P* = 0.031). Similarly, the researchers claimed that the passive muscle tension from the plantar flexor increased.

In a previous study [54], the mel frequency cepstral coefficient (MFCC) and ${\mathrm{MMG}}_{\mathrm{RMS}}$ were used to train an SVM classifier for the identification of fatigued and non-fatigued RF, VL, and vastus medialis in SCI. MFCC were then obtained as its time-localized frequency information using short-time Fourier transform of a stationary signal with a window frame of 25 ms. The MFCC features exhibited 90.7% accuracy, whereas the RMS feature only showed 74.5% accuracy.

Meanwhile, three studies [55–57] have also examined muscle fatigue via analyzing torque and MMG profiles. One of these studies [55] assessed the MMG responses from the quadriceps muscles to quantify its changes between pre- and post-fatiguing conditions in SCI patients in NMES-evoked cycling. The peak torque and normalized ${\mathrm{MMG}}_{\mathrm{RMS}}$ and ${\mathrm{MMG}}_{\mathrm{MPF}}$ values showed significant trends between pre- and postelectrical excitation for individual muscles. The researchers also observed a change in the MMG signals as a function of the cycling time for the VL, RF, and VM muscles. Typically, ${\mathrm{MMG}}_{\mathrm{RMS}}$ and epT significantly (*P* < 0.05) decrease as the leg cycling exercise increases for the quadriceps groups.

On the other hand, Massimiliano Gobbo and colleagues [57] have investigated the validity of MMG to assess the fatigue that develops during NMES in rehabilitation services. After a fatiguing stimulation of 50 single twitch and 2 Hz for 25 s, a correlation was found between the mean values of normalized ${p}^{\mathrm{T}}$% and ${\mathrm{MMG}}_{\mathrm{PP}}$ %. It was further reported that ${p}^{\mathrm{T}}$ % and ${\mathrm{MMG}}_{\mathrm{PP}}$ % decreased from 100% of their initial values to 50 and 60% for the BB, respectively, and to 43 and 47% for the VL, respectively. In addition, the decreases in ${p}^{\mathrm{T}}$% and ${\mathrm{MMG}}_{\mathrm{PP}}$ % exhibited a different linear correlation. Thus, the introduction of electrical activation of skeletal muscles and MMG responses into assistive technology that involves analysis of torque of a particular muscle remains to be unclear.

Under both artificial electro-muscle excitation and MVC, Jo et al. [56] examined the fatigue from the ankle joint and performed a torque analysis. The raw MMG data were then extracted during 30 min of repeated stimulation of the tibialis anterior (TA) muscle, sampled at 1 kHz and bandpass filtered at 8–100 Hz. The amplitude, i.e., convex-hull, peak-to-peak, and Lempel–Ziv algorithm, and median and mean frequency-based MMG features were also utilized for the quantification of fatigue of the TA muscle. The ankle joint torque has been used to analyze fatigue after low-pass filtering of the raw torque at a cutoff frequency of 3 Hz. The muscle fatigue was further indicated by a linear decrease in torque during consecutive stimulation patterns. The coefficient of determination $\left({r}^{2}\right)$ was 0.7823. However, it was noted that the frequency components of MMG signals demonstrated a weak linear relationship with fatigue. Thus, the use of the stimulation protocol relevant to NMES popular in clinical settings supports the finding that the technique may fit the design of electrical muscle excitation feedback control system, which is useful in assisting week/injured muscles.

Four studies [58–61] have been identified to discuss the physiological behaviors after fatigue administration. In one of these studies [58], the authors evaluated the correlation between the temporal and spectral features of MMG from 10 SCI and 10 health volunteers undergoing recovery by NMES.${\mathrm{MMG}}_{\mathrm{RMS}}$ and ${\mathrm{MMG}}_{\mathrm{MF}}$ from the analysis of window length were examined. An increase in RMS and a decrease in MF were then observed from both groups. The authors concluded that the temporal features might originate from the increased amplitude of mechanical wave from motor unit coherence, whereas the decrease in MF might be attributed to motor unit adaptation.

On the other hand, two studies [59, 61] evaluated the effects of stretching on mechanical and electrical responses, wherein both analyses found constant electrical parameters but a divergence in the mechanical features. One of these studies [59] used a stretching protocol in order to evaluate the changes in the mechanical and electrical properties of EMG and MMG on GM. Significant decreases in the RMS and MF of EMG have been noted, and a conduction velocity was observed after the fatiguing protocol. Similarly, the RMS and peak-to-peak values of MMG were noted to decrease, whereas the speed of electromechanical impulse and the ½T_r_ increased. The authors claimed that all RMS values of EMG and MMG returned to their pre-fatigued values after stretching, whereas the peak rate of force development, derived acceleration, and the ½T_r_ showed further reductions. This led the researchers to a conclusion that the application of acute stretching to previously fatigued muscles weakened the mechanical but not the electrical properties.

In contrast, another study [61] has evaluated the electrical and mechanical manifestations of muscles after a set of six electrical stimulations before and after stretching. The findings are in line with those obtained in the above-mentioned study [59], revealing no significant changes in the EMG signal parameters. In addition, the study found reductions in the force acceleration, peak force, and peak-to-peak MMG values, and these reductions were determined to be accompanied by a possible concomitant decrease in muscle stiffness. Moreover, the researchers found a poor correlation between the MMG and force parameters, wherein a bout of acute passive stretching alters the mechanical but not the electrical parameters of electrically stimulated muscles.

Furthermore, the mechanical properties of skeletal muscles were also investigated in a previous study [60], with the aim of characterizing the architectural changes in the human MG under interpolation conditions. Using ultrasound images obtained from electrically excited MG under plantar flexion at 20, 40, 60, 80, and 100% MVC, the researchers found that superimposed MMG amplitudes exhibited a curvilinear decrease accompanied by a fascicle shortening, with increases in the contraction intensity up to 100% MVC. In contrast, the study argued that the superimposed twitch amplitude decreased linearly with increases in the contraction intensity up to 80% MVC.

Four studies [62–65] have also reported on the use of MMG and fatigue as a reflection of muscle endurance. One study [62] aimed to diagnose the drawback of exercise-based improvements in disabled individuals. Assisted by electrical muscle activation, the walking speed and muscle endurance were compared to the working function and strength. As per the endurance index, the endurance was noted to increase with increases in the walking function in individuals with multiple sclerosis. Similarly, another study [63] highlighted the dependency of the endurance index on MMG recorded from electrical activation of the erector spinae muscle compared with those from the voluntary or muscle oxygen levels. Another study [64] has also used different stimulation protocols to examine endurance in SCI-paralyzed patients under different workloads. The authors reported that a high stimulation intensity, compared with the lower intensity, can lead to increased muscle fatigue and hypothesized that the standard co-activation stimulation protocols are less prone to cycling endurance time accounted in NMES-based functional recovery compared with alternative stimulation.

Meanwhile, a previous study [65] evaluated the endurance, feeling of fatigue, and mitochondria capacity using NMES, near-infrared spectroscopy (NIRS), and MMG at different age ranges. The forearm flexors of 10 abled bodies and 16 Friedreich’s ataxia (FRDA) were electrically stimulated for 3 min at 2, 4, and 6 Hz, with a resting interval of 5 s. The MMG records indicated a considerable correlation in terms of mitochondrial capacity, disease severity, and muscle-specific endurance in FRDA. Based on these findings, it can be concluded that the disease frequency and progression in the FRDA population can be monitored.

It was indicated that MMG signals are obtained from muscles, which represent the different physiological conditions of those muscles through fatigue protocols. It is likely that a decrease in spectral component during functional recovery produced by NMES can be due to motor unit adaptation, whereas the increase in MMG_RMS_ might be due to motor unit coherence. In addition, a decline in MMG parameters and torque with an increase in fatiguing NMES demonstrates the usefulness of electrical stimulation and muscle mechanics in determining muscle injury in SCI population. Hence, fluctuation in mechanical features manifested as a muscle response in different experiments requires further exploration. It was also pointed out that LFF might be attributed to low % MVC. However, this was contradictory to other studies as this was identified to be due to the slower ability of muscles to recover their normal activity under low frequency or overlap between signals from damaged and fatigued muscles. The role of muscles mechanics has also been determined using MFCC, which is a feature extraction technique that uses speech signal processing and other nonstationary signals such as EMG classifications and electroencephalography (EEG) [66]. As detailed in the literature, the developed model exhibited good accuracy in muscle fatigue assessment. The features from MMG have also reported the correlations among specific muscle endurance, mitochondrial capacity, and diseases severity in FRDA population.

Collectively, the findings from these studies provide evidence that muscle fatigue should be re-investigated. Specifically, the overlap between muscle damage and fatigue, the decrease in the angle caused by muscle weakness, and the overlap between muscle endurance and disease severity and progression in populations other than the FRDA population should be further examined. Thus, muscle mechanics based on MMG has been experimented in electrical stimulation-based cycling exercises but requires further investigation in biofeedback control system required in assistive technology for the limbs rehabilitation.

### Force assessment

As presented in Table 7, four studies [67–70] have been determined to examine the influence of muscle conditions on force and MMG recorded under NMES. One of these studies [69] has attempted to determine the effect of temperature on contractile parameters using temporal and spectral features of MMG signals. After electrical intensity was delivered to the GM and SOL muscles, the force signals and MMG responses were examined under a controlled temperature. The authors of this study then reported a decrease in the maximum peak force development and relaxation under cooling conditions. In contrast, the CT and ½T_r_ increased under hypothermia. Further, an increase in M-waves was also noted, which justifies the decreases in the muscle conduction velocity. These findings encourage the use of MMG for screening the muscular contractile behaviors under varying physiological circumstances. The use of MMG-, EMG-, and force-based approaches to evaluate the time course of stretching-induced changes in the mechanical and viscoelastic properties of MTU was examined in [67, 68]. The findings obtained in one of these studies [67] revealed that an increase in the EMD followed a reduction in ${p}^{\mathrm{F}}$. The time delay between EMG and MMG recovered to its initial value within 15 min, whereas the delay between MMG and force value was maintained for 2 h. These findings only confirmed the effect of stretching and encouraged the use of MMG, EMG, and force as indicators of the recovery of muscle properties after the induction of mechanical stress on the MTU viscoelastic properties. On the one hand, ${p}^{\mathrm{F}}$ and ${\mathrm{MMG}}_{\mathrm{PP}}$ did not exhibit recovery, and on the other hand, the short recovery observed in ${\mathrm{MMG}}_{\mathrm{RMS}}$ indicated a recovery in the viscoelastic properties of parallel components after a short period [68]. Biomechanical and physiological responses were also used to evaluate the slowing of fatigue due to changes induced by occupational mechanical exposure. Using various force amplitudes, another study [70] applied electrical stimulation quantities to the triceps brachii (TB) at 15% MVC to elicit LFF. Therefore, the subject performed MVC for 5 s, and the force was recorded for 3 s. The authors then concluded that (i) local fatigue might be reduced in individuals performing low-load tasks and (ii) muscle rest does not exert more marked effects than those predicted by variations in the force amplitude. These findings imply that changes in mechanical action with or without rest delay can reduce the rate of fatigue development compared with the development observed under isometric, isotonic, and sustained conditions. Therefore, it was suggested that muscle exposure to time-varying forces might reduce the degree of local fatigue during low-load tasks. The observations described in this review highlighted the relationship of muscle conditions, force, and daily tasks using MMG through NMES. Hence, muscles screening should take physiological conditions and daily occupational exposure circumstances into consideration.

**Table 7:** Overview of studies of force and MMG

Authors	Sensor and electrode type	Electrode site	Dataset	Methodology	Results and discussion
Study 1: analysis of the effect of temperature on MMG and the force response					
[69]	EMG: Ag–AgCl (diameter = 8 mm, inter-electrode distance = 35 mm); MMG: microphone sensor (Daia Medical, Tokyo, Japan; diameter = 10 mm, mass = 5 g); Force: (model LU-00KSB34D, Kyowa, Tokyo, Japan)	SOL and MG	8 healthy male subjects, age (means ± SEs) 23.3 ± 0.5 years, height 173.6 ± 2.7 cm, body mass 71.1 ± 4.6 kg	At temperatures of 34, 15, 20 and 25 °C, a 10-Hz stimulation for 8 s with a 30-s rest between trials was delivered	Under cooling conditions, the CT and ½T_r_ increased, and the maximum peak force development ($dF/dt$) and relaxation ($RdF/dt$) decreased; in addition, a low RMS of the force fluctuations and low MG and SOL MMG amplitudes were obtained
Remark: MMG can be used to study the muscle contractile properties under a wide range of physiological conditions					
Study 2: analysis of the effect of changes in contractile and viscoelastic properties due to EMD					
[67]	MMG: accelerometer (model ADXL202JE, Analogue Devices, Norwood, MA, USA); EMG: four silver bar electrodes; Force: (model SM-200 N, Interface, UK; operation range = 0 and 200 N)	MG	16 healthy male subjects, age 24 years, body mass 75 ± 2 kg, stature 179 ± 2 cm	A supramaximal stimulation followed by a rest time of 10 min was delivered while the ankle was positioned at 20°. Before and after stretching, a set of three tetanic stimulations was delivered and followed by 15 min to 2 h s of rest	During recovery, the delay between EMG-MMG returned to pre-stretching values within 900 s, and Δt MMG-F remain lengthened for 7200 s
Remark: significant lengthening of the EMD was observed, possibly due to changes in the MTU stiffness					
Future works:					
1. The involvement of parallel and series elastic components in the lengthening of ∆t MMG-F after stretching should be examined					
2. The sites and duration of the MTU deformation after stretching should be analyzed					
3. The release of ${Ca}^{2+}$ and the involvement of sensitivity in the stretching-induced changes in excitation–contraction coupling should be investigated					
Study 3: analysis of the use of MMG, EMG and force approaches to evaluate the time course of stretching-induced changes in mechanical and viscoelastic properties of MTUs					
[68]	MMG: mono-directional accelerometer (model ADXL202JE, Analogue Devices, Norwood, MA, USA); EMG: three Ag–AgCl electrodes	MG	11 healthy male subjects, age (means ± SDs) 22 ± 1 years, body mass 77 ± 5 kg, stature 1.78 ± 0.05 m	Stimulation with an amplitude of 10–100 mA, a pulse duration of 307 µA, and lasting for 5 s to 1 min at 50 Hz was followed by 110% supramaximal stimulation and rest for 600 s. A tetanic stimulation was delivered every 5 s for 15 min and followed by 2 h of rest	The MMG-RMS recovered to the pre-stretching values, and the MMG-PP and ${P}^{F}$ values remained low. No difference in the EMG response was found between the two experiments (*p* > 0.05)
Remark: no significant differences in the control parameters for the EMG, MMG and force features were found during the stimulation sets and during the 2-h recovery period					
Future work: the rule of transverse MTUs based on the MMG amplitude should be studied					
Study 4: analysis of the effects of the amplitudes in the force variation between force and local fatigue using biomechanics and physiological measures					
[70]	ES: Grass model S48; EMG: bipolar surface electrodes (Ag–AgCl electrodes, Ambu Blue Sensor N, Denmark); MMG: (Bruel and Kjaer 4507 ± 70 g)	TB	15 healthy male subjects, age 24 ± 4 years, mean height 177.7 ± 4.9 cm, weight 75.8 ± 8.7 kg	EMG, MMG, and blood flow velocity recordings of the triceps were collected at 2-min intervals during supramaximal fatiguing stimulation (20 and 100 Hz, pulse duration of 50 µs and train duration of 1 s) and at baseline. Test contraction at 15% of the force was exerted for 12 s	Changes in mechanical action with or without rest delayed and reduced the rate of fatigue development compared with those observed under isometric, isotonic and sustained conditions
Remark: force variation at different amplitudes might yield slower fatigue responses under a set time course than those observed during sustained low-level contractions					

### Assessment of muscle stiffness

Using MMG and NMES, six studies [71–76] in Table 8 have been identified to examine muscle stiffness. One of these studies [75] has utilized evoked MMG signals from voluntary MMG and walking acceleration in order to estimate stiffness. Combined with the undamped natural frequency, the mass of the VL estimated as 0.99% of the subject’s weight was also used in an attempt to estimate stiffness. The result was then compared with the stiffness estimated using the identification technique, wherein muscle stiffness was found to exhibit a prior relationship with both workload and power.

**Table 8:** Overview of the use of ES and MMG on the assessment of muscle stiffness

Authors	Sensor and electrode type	Electrode site	Dataset	Methodology	Results and discussion
Study 1: analysis of muscle stiffness at various workloads, cadences, and power levels					
[75]	MMG: capacitor microphone (MXE-4758; Primo, Tokyo, Japan); ES: 10-mm diameter Ag–AgCl (Vitrode J-150; Nihon Kohden)	VL	8 able subjects, age 21–24 years	The subjects pedaled an electrically braked ergometer set to 47 W. Stimulation pulses were delivered at 30° with 3-, 2- and 1.5-s inter-pulse intervals and a constant power of 40, 60, and 80, respectively	With the knee angle set to 80°, the muscle stiffness was in direct proportion to the workload and power
Remark: the muscle stiffness progressively increased with increases in the pedaling rate					
Future work: the effect of non-active and active muscles on the viscous coefficient and the muscle response after the application of various phases per cycle should be investigated					
Study 2: estimation of the muscle stiffness from evoked MMG					
[71]	ES: Ag–AgCl electrodes (F-150S; Nihon Kohden); DMMG: capacitor microphone (MX-E4758; Primo, Tokyo, Japan; weight = 0.78 kg, sensitivity = − 43 ± 3 dB at 1 kHz, gain = 0.5 to 4000 Hz); AMMG: (MP-110–10-101, MediSens, Saitama, Japan)	TA	7 healthy male subjects, aged 22–24 years	A monopolar pulse with a 500-µs width and an inter-pulse interval of 1 s was applied in each trial and repeated five times	No significance difference was found between the natural frequencies from the velocity and displacement obtained by MMG
Remark: the velocity component measured with a differential circuit reached a steady state value within a short time, and motion is thus recommended					
Future work: the transverse muscle stiffness should be further estimated					
Study 3: analysis of muscle strength					
[74]	MMGs: capacitor microphone (MX-E4758; Primo, Tokyo, Japan); ES: Ag–AgCl surface electrodes (F-150S, Nihon Kohen)	GM	8 healthy male subjects, age 22–24 years	The subject was asked to pedal at speed of 3 km/h with a gait cycle of 1.8, 1.5, 1.2, and 1.1 s, and a monopolar rectangular pulse width of 500 µs and an amplitude of 20-mA was applied	A progressive increase in muscle stiffness was detected with increases in the pedaling rate
Remark: the stable viscous coefficient was attributed to the friction effect from active and non-active fibers					
Future work: the cause of the stable viscous coefficients should be investigated					
Study 4: analysis of the stiffness of the gastrocnemius muscle and the waking speed					
[73]	ES: Ag–AgCl (F-150S, Nihon Kohden); MMG: capacitor microphone (MX-E4758; Primo, Tokyo, Japan)	GM	8 healthy male subjects, age 22–24 years	Stimulation for 500 µs with a 20-mA amplitude was delivered to the GM while the subject walked on a treadmill at 2, 3,4, 5 km/h with a gait cycle of 1.8, 1.5, 1.2, and 1.1 s	The stiffness was indicated by an increase in the natural frequencies f1 and f2, which increased with increases in the gait speed
Remark: a constant natural frequency f3 is likely caused by subcutaneous tissue					
Future work: the natural frequency corresponding to the soleus muscle should be further studied, and the natural frequency of GM should be clarified					
Study 5: analysis of the effect of static stretching (SS) on joint angle stiffness					
[72]	EMG: silver bars (model ELSCH004, diameter = 1 mm, length = 10 mm, inter-electrode distance = 10 mm, OT Bioelettronica, Turin, Italy); MMG: accelerometer (model ADXL202JE, Analogue Devices, Norwood, MA, USA); force: load cell (model SM-2000 N, Interface, UK; operation 0–2000 N)	GM and GL	19 healthy male subjects, age (mean ± SD) 24 ± 3 years, body mass 76.4 ± 8.9 kg, stature 1.78 ± 0.09 m	MMG, EMG and force signals were recorded before and after SS. The joint stiffness and the force from plantar flexors were measured after a supramaximal + 10% tetanic stimulation (3 s, 50 Hz, 10–100 mA, and 304 µs) applied at 0°, 10° and 20°	The SS and joint stiffness increased with increases in the joint angle
Remark: the increase in dorsiflexion with ${\mathrm{R}-\mathrm{Delay}}_{\mathrm{TOT}}$ might be due to joint stiffness					

Furthermore, the analysis on the synchronous averaging of evoked MMGs in a previous study [71] was performed through identifying both longitudinal and transverse muscle stiffness from MMG signals obtained via electrical activation of the peroneal nerve. A capacitor microphone and a differential circuit were used for recording purposes. The stiffness was measured from a natural frequency, assuming that the mass of the TA muscle equals 0.2% of the participant’s weight. In another study [76], the singular value decomposition (SVD) was used in determining the transfer function from stimulated to evoked MMG signals. The velocity MMG yielded two natural frequency components denoted as f1 and f2: f1 mirrored the longitudinal stiffness, which was approximated to the values in the displacement and acceleration MMG systems, and f2 was determined from the velocity MMG and was found to be similar to the natural frequency of the acceleration MMG system. The resulting value was then regarded as the transverse muscle stiffness but was not estimated from MMG signals. The displacement and acceleration MMG yielded three natural frequencies, which were denoted as f1, f2, and f3, and no significant difference was determined between the natural frequencies from velocity and displacement MMG.

In the same study [76], electrical stimuli were transcutaneously delivered to the VL muscle while the participants were ask to pedal an ergometer. The mass of the muscle and the coefficient of the transfer function estimated using SVD, which was performed as described in another previous study [71], were used in order to estimate the muscle stiffness and viscous characteristics under low power output [74]. The authors then observed a progressive increase in muscle stiffness as the pedal rate also increased. The stiffness was approximated at *P* < 0.025, with a decision coefficient of 0.984. However, the viscous coefficient was determined to not increase. A possible reason for these findings is the rubbing between active and inactive muscle fibers due to the force exerted in direct proportion to the velocity. Clearly, this study has examined the muscle activity using EMG and found that ${\mathrm{EMG}}_{\mathrm{RMS}}$ showed changes at different pedaling rates in various subjects. In contrast, similar mean ${\mathrm{EMG}}_{\mathrm{RMS}}$ values were found at 40, 60, and 80 rpm. Thus, future studies should elucidate the mechanism underlying the associations among muscle stiffness, viscous coefficients, and muscle function.

More recently, another study [73] has examined the stiffness obtained based on the GM dependency on gait speed. During treadmill training, stimulated and nonstimulated MMGs were recorded from the GM, and the evoked MMG signals were then used to identify the stiffness index based on the corresponding natural frequencies. The researchers reported that the frequencies and thus the stiffness increased as the gait speed increases.

The authors of a previous study [59] have also used changes in the joint angle torque to estimate stiffness. The responses from electrical excitation of the GM, namely, the EMG, MMG, and torque, were recorded to estimate the muscle stiffness before and after static stretching. After dividing the information into electrochemical and mechanical components, the offline ${\mathrm{R}-\mathrm{Delay}}_{\mathrm{TOT}}$ was determined based on the force, EMG, and MMG signals recorded at ${P}^{\mathrm{T}}$. A decrease in the main electromechanical component was then observed as the joint angle increased before and after static stretching. This study has also determined that during muscle relaxation, an increased dorsiflexion angle could be attributed to joint stiffness rather than the electrochemical process.

Authors of this study have further demonstrated that the anthropometric features and geometry of the limbs can contribute to joint stiffness and found that the frequency components increased as stiffness increases [76], even though the so-called viscous coefficient did not seem to vary. In addition, the changes in the joint angle torque after stretching tasks have exhibited a progressive decrease in electromechanical delay components with increases in the joint angle at pT [72]. As what has been discussed in a previous study [77], the effects of inactive and active muscles on the viscous coefficient [75], subcutaneous tissue [73], MTU stiffness, and joint angles should be considered in further studies [78]. It can be concluded that the anthropometric, electrochemical, and mechanical components, daily physical tasks, and geometry of the muscles are of importance in terms of estimating for muscle stiffness using myographic signals.

### Torque assessment

As presented in Table 9, nine studies [79–87] have reported that torque is mirrored by the MMG features from electrically contracted muscles. The authors of one of these previous studies [79] used a combination of MMG and torque signals in determining the dependency of the contractile properties of a muscle on its length. The BB was electrically stimulated using 10- and 30-Hz fused and unfused tetanic stimulation, and then, evoked flexion torque was measured at six joint angles ranging from 75° to 150° at 15° increments. The RMS values of both the torque and MMG signals obtained with different stimulation frequencies confirmed the reliability of MMG for evaluating the contractile properties of muscles. In addition, the findings have demonstrated that the torque fluctuates with variations in the muscle length (Table 10).

**Table 9:** MMG and muscle torque

Study 1: analysis of torque and MMG fluctuations at various joint angles					
Authors	Sensor and electrode type	Electrode site	Dataset	Methodology	Results and discussion
[79]	Torque: strain gauge (model LURA-100NSA1, Kyowa Electronic Instruments, Japan); MMG: microphone sensor (QTEC, Japan; diameter = 8 mm, mass = 5 g)	BB	9 healthy male subjects, age (mean ± SE) 24.5 ± 1.1 years, height 171.3 ± 2.8 cm, weight 68.1 ± 2.6 kg	A 10 Hz, 5-s train at 30 Hz was applied at each angle to familiarize the subject with the pain induced by the tetanic stimulation. The elicited torque at 75, 90, 120, 135 and 150° was measured and was analyzed based on recorded MMG signals. A 100-µs pulse duration was provided to ensure an increase in the muscle contraction with torque	The MMG amplitude was correlated with the CT and the ½T_r_ and reflected the changes in torque relaxation observed with increases in the muscle length
Remark: the correlation of the MMG amplitude with the half-relaxation and contraction times indicates that MMG is a valid tool for monitoring changes in the contraction features of skeletal muscle					
Study 2: analysis of the effect of age and muscle mechanics					
[87]	MMG: 9-mm square with a thickness of 4.5 mm and a mass of 0.75 g (MP110-10–101; MEDiSENS, Tokyo, Japan; sensitivity = 500 mV/g); EMG: (1.5—1 cm; Kendall-LTP, Chicopee, MA, USA)	TA	10 young male subjects, age 27.1 ± 3.8 years (range 21–33 years), height 174.2 ± 7.7 cm, weight 78.7 ± 7.8 kg; 10 old male subjects, age 79.0 ± 2.5 years (range 75–83 years), height 171.4 ± 5.7 cm, weight 79.2 ± 10.2 kg	The M-wave was detected after delivering a 400-V pulse with a width of 50 µs and a current intensity of 70–150 mA. The pT was assessed after a supramaximal twitch and a 30–60-s rest	The electrical and mechanical features of muscles that are either electrically or voluntary induced are affected in different manners by ageing
Remark: the MMG and EMG patterns presented similar shapes in young subjects and were altered in the old population					
Study 3: analysis of muscle features during long and short stimulation protocols					
[80]	MMG: laser-distance sensor (M5 L/20, MEL Mikroelektronik, Germany, range = ± 10 mm, sensitivity = 1 V/mm, linearity = 0.6%, resolution < 6 µm, bandwidth = 0–10 kHz); EMG: two bar electrodes (1 cm × 1 mm × 1 mm); force: load cell (Interface, model SM-100 N, operating range = 0–100 N)	TA	14 healthy male subjects, age 20–35 years	12.5-s short duration pulses (0.4, 6.0, 1.0, 4.5, 1.8, 3.0, and 2.5 Hz) and 6-s pulses (0.4, 0.6, 0.8, 1.0, 1.2, 1.4, 1.6, 1.8, 2.0, 2.5, 3.0, 4.0, 5.0, and 6.0 Hz) with a 300-s rest between pulses to avoid fatigue	A similar transfer function from the torque and laser-detected MMG signal resulted in a decline in the sinusoidal amplitude and a phase shift in the torque and MMG
Remark: the torque and laser MMG recordings yielded similar transfer functions, which validates the use of MMG for screening the mechanical properties of muscle tendons units					
Future work: elicitation of the whole motor nerve can be used to investigate the shortening of non-active muscle fibers					
Study 4: model of human muscle using a triangular frequency and an amplitude train					
[81]	MMG: laser distance sensor (M5 L/20, MEL Mikroelektronik, Germany, range = ± 10 mm, bandwidth = 0–10 kHz, linearity = 0.6%); EMG: pair of silver bar electrodes; force: load cell (model SM-100 N, Interface Inc, Scottsdale, AZ, USA; operation range = 0–100 N)	TA	10 healthy male subjects, age 23–35 years	Increase from 2 to 35 Hz over 7.5 s or decrease from 35 to 2 Hz over 7.5 s; the amplitude was increased from V_min_ to V_max_ and decreased from V_max_ to V_min_. The mean torque, MMG and EMG for each 5% in the ∆ frequency range of 2 Hz (0%) to 35 (100%) Hz were used to determine the stimulation frequency and additional muscle torque	Under DGR, the amplitude and frequency triangle are in line with the additional torque and muscle displacement
Remark: bypassing the CNS, the additional torque and MMG are induced by the intrinsic muscle property					
Future work: the number of subjects needs to be increased to validate the EMG behaviors in DGR and UGR induced by a frequency triangle					
Study 5: analysis of contractile parameters from the TMG and the torque twitch response in human VL muscle					
[82]	TMG: displacement sensor (G40 digital-optical comparator, TMG-BM Ltd., Slovenia); force: transducer (TSD121C, BIOPAC Systems Inc., USA)	VL	19 healthy male subjects, age 46.1 ± 17.8, body mass 78.4 ± 12.7 kg, height 1.74 ± 0.05 m, BMI 26.0 ± 4.2 kg/m^2^	1-ms pulse current separated by 10 s from the motor threshold to the maximum stimulation current at incremental steps of 5 mA, with a maximum stimulation amplitude of 10–100 mA	Based on TMG, the force inter-method correlation was significant with Ts and Tr but not significant with other parameters
Remark: the TMG-measured contractile parameters are shorter during the contraction phase due to their dependence on intrinsic muscle properties					
Study 6: prediction of NMES-evoked knee torque using SVR					
[83]	ES: 9 × 15-cm^2^ self-adhesive electrodes (Hasomed GmbH, D 39114, Magdeburg, Germany); MMG: accelerometer (Sonostics BPS-II VMG transducer, sensitivity = 30 V/g)	RF	8 healthy male subjects, age 23.4 ± 1.3 years, body mass 70.4 ± 5.8 kg, height 1.72 ± 0.05 m	Square-wave 30-Hz pulses with a 400-µs duration were applied; the current amplitude was increased from 20 to 80 mA over a duration of 48 h, and a recovery time of 10 min was established after each trial	A high prediction accuracy with a coefficient of determination of ${R}^{2}=94\%-89\%$ and a low root mean square error of 9.48–12.95 was obtained
Remark: the study was limited to healthy volunteers					
Future work: the model should be examined with disabled subjects, and normal and fatigued knee extensors during standing or itinerant tasks should be classified					
Study 7: analysis of twitch torque and reliability of recruitment curves					
[84]	ES: self-adhering stimulating electrodes (1 × 1 cm); MMG: accelerometer (EGAS-FS-10-/VO5, Measurement Specialties Inc., Hampton, VA, USA)	Soleus	16 subjects, age 23.5 ± 1.9 years, body mass 71.1 ± 10.6 kg, height 173.4 ± 8.0 cm	A pulse (1 ms, 100–400 V, and 2–100 mA) was administered with increments of 0.5–2 mA to detect the maximum H-reflex and decreases in the H-reflex. Thereafter, an increment of 2–5 mA was applied to localize the plateau in the M-wave amplitude; a rest period of 3 to 5 s was applied between stimuli, and four to seven stimuli were included in each individual pulse	A strong test–retest reliability with *p* > 0.05 was found over days. A low coefficient of variation for MMG_max_ with the maximal stimulation intensity and torque was found
Remark: a new parameter, M + H, exhibited a strong correlation with the twitch torque and MMG					
Future works:					
1. The gross lateral movement of muscles under voluntary contractions should be investigated					
2. Comparisons of M-wave and H-reflex should be performed, and features related to the soleus and gastrocnemius muscles after treatment should be investigated					
Study 8: system identification of muscle with parallel fibers					
[85]	ES: Ag–AgCl surface electrodes (F-150S, Nihon Kohden, Tokyo); DMMG: capacitor microphone (MX-E4758, Primo, Tokyo); AMMG: MP-110–10-101 (MediSens, Saitama); Force: FlexForce A201-1 (Nitta, Osaka)	Abductor pollicis brevis	6 healthy male subjects, age 21–25 years	The median nerve was electrically stimulated with a 500-µs rectangular pulse at an interval of 1 s and a bandwidth of -3Db; the pulse was repeated 20 times	Muscles with parallel fibers can be modeled by a 6^th^-order model using system identification of DMMG and AMMG
Remark: the abductor pollicis brevis presented a higher frequency than the tibialis anterior, which might be due to the anatomical structure					
Future work: the effect of the anatomical properties of the tibialis anterior and abductor pollicis brevis on the natural frequency should be verified					
Study 9: prediction of the muscle torque using ANN					
[86]	Torque: dynamometer (Biodex Medical system Shirley, NY, USA); ES: 9 × 15-mm^2^ self-adhesive electrodes; MMG: transducer (Sonostics BPS-II VMG, sensitivity = 30 V/g)	Right and left GM	8 SCI volunteers with ISNCSCI classes A and B, 3 for training and 5 for testing	30 Hz, 200-µs duration and 100-mA amplitude; the MMGrms, MMG ZC and torque were recorded with a knee angle of 30°	The ANN-based prediction using MMG and dynamometer recordings were close with a *p*-value of 0.33
Remark: MMG-RMS-ZC showed higher prediction than RMS alone					
Future work: other MMG features and other ANN models should be investigated

**Table 10:** Miscellaneous (validity for clinical monitoring)

Authors	Sensor and electrode type	Electrode site	Dataset	Methodology	Results
Study 1: analysis of surgical monitoring					
[92]	MMG: (SentioMMG, Sentio, LLC, Wixom, MI, USA)	VM, TA, BF and gastrocsoleus	15 subjects with herniated nucleus pulposus (HNP), 31 subjects with lateral recess stenosis	The stimulation current was measured beyond 1 mA after passing a ball-tripped sterile probe onto the surgical field. MMG signals were recorded to indicate the lowest current at which a motor action potential was measured. The stimulation current was increased to evoke MMG	The affected nerve root maintained a stimulus threshold of 1 mA following decompression but exhibited an increased MMG amplitude
Remark: the method is adequate fir root decompression and provides direct feedback to the surgeon					
Future work: the threshold change among patients with acute and chronic impingement should be studied, and the valuable measurements obtained from advanced IONM MMG for quantifying changes in health pre- and post-decompression and their relationship to post-operative clinical outcomes should be investigated					
Study 2: analysis of neuromuscular monitoring					
[89]	MMG: piezo-electric (diameter = 1.6 cm, model 1010, Grass Instruments, Astro-Med, Inc., West Warwick, RI, USA; frequency = 2.5 Hz to 5 kHz, output = 20–40 mV)	Adductor pollicis and VM	4 male and 8 female subjects, age 47 ± 21 years, weight 75 ± 8 kg, height 165 ± 12 cm	Mivacurium was injected for 5 min after the ulnar nerve and IM branches of the femoral nerve were supramaximally stimulated for 12 s with a 70-mA maximum current using a constant current stimulator. Acoustic signals were recorded from both muscles	The control twitch amplitude of the adductor pollicis was more significant than that of the VM
Remark: the VM was associated with a shorter onset time, a less pronounced maximal effect and more rapid recovery of the NMB compared with the adductor pollicis					
Study 3: comparison of KMG and EMG for clinical monitoring					
[90]	EMG: ${\mathrm{Datex}}^{\mathrm{TM}}$ electrosensor; KMG: KMG sensor (Datex M-NMT MechanoSensor™)	Adductor pollicis	27 female subjects, age 18–65 years	NMB was measured after injection of anesthesia, and NMT was measured after supramaximal stimulation for 5 min with 5-s pulses at 50 Hz; the Bland–Atman method was used to determine the difference between MMG and EMG	KMG overestimated the EMG due to variation from 65 to 100%, and these sensors cannot be used interchangeably
Remark: KMG and EMG sensors cannot be used interchangeably					
Future work: the use of KMG for monitoring a clinical endpoint should be verified					
Study 4: analysis of diaphragm contractility using MMG					
[91]	ES: (Neuropack MEB-9100, Nihon Kohden Inc., Tokyo, Japan); MMG: accelerometer (MPS110, MediSens Inc., Saitama, Japan)	Right phrenic nerve	21 young subjects, age 22.5 ± 3.2, height 165.3 ± 78.6 m, weight 56.5 ± 8.1 kg; and 20 elderly subjects, non-smokers, age 70.9 ± 4.4 years, height 157.9 ± 8.2 m, weight 53.4 ± 8.5 kg, smokers, age 74.2 ± 5.9 years, height 154.8 ± 9.1 m, weight 51.4 ± 9.1 kg	The MMG signals from a contractile diaphragm were recorded after stimulation of the phrenic nerve. The correlations between diaphragmatic MMGs and respiratory parameters were assessed using Pearson correlation coefficients (significant if *p* < 0.05)	The correlations between diaphragmatic MMGs and respiratory parameters were significant (*p* < 0.05)
Remark: diaphragmatic MMG was strongly correlated with inspiratory muscle strength and might reflect the diaphragmatic contractility more directly and with higher sensitivity than the conventional method					

Some researchers [87] have examined the effects of aging on muscles through a comparison of MMG and EMG features with torque’s profiles. The muscles of old and young individuals were subjected to electromyostimulation before and after 10 s and then less than 100% MVC, wherein both MMG and ${P}^{\mathrm{T}}$ were observed to have been significantly increased, but the M-waves remained steady. However, no effect of aging was detected. During voluntary contraction, the MMG and EMG profiles in young and old men exhibited similar shapes, which indicated that the electrical and mechanical properties of voluntary or artificial stimulation of muscles exhibited different behaviors at different ages.

Many studies have used a combination of MMG and torque signals in an effort to characterize muscle mechanics. Using short and long stimulation pulses, the detected MMG and torque signals yielded two transfer functions, and these functions were analyzed to characterize the TA muscle joints. After stimulation intensity was delivered, the torque was determined offline based on the tension recorded from the foot and ankle lever arm. Therefore, the in vivo mechanical properties of muscle joints obtained using short stimulation protocols were obtained from MMG and torque signals [80]. An analysis of the torque, MMG, and EMG signals obtained under NMES with triangular trains with varying frequencies and amplitudes has yielded the muscle input/output relationship of the TA under static contraction and thus showed the module of muscle force production [81]. The relationship between the stimulation frequencies yielded the average torque, MMG, and EMG signals for each 5% of the ∆ frequency range from 2 Hz (0%) to 35 Hz (100%). As per experimental results, an additional torque corresponds to an increase in the extra-muscle displacement (MMG) during down-going ramp (DGR) rather than up-going ramp (UGR) for both frequency and amplitude triangles. In addition, the authors suggest that stimulation parameters close to physiological quantities indicate the influence of intrinsic muscle factors on additional torque and MMG signals.

However, the findings obtained in a previous study [82] have been deemed controversial. The longitudinal torque and transverse TMG have yielded differences in the contractile parameters of the VL. Therefore, these findings indicate that the TMG mechanical responses can reflect the intrinsic contractile parameters of skeletal muscles.

A study conducted a year later [83] used computational intelligence to solve the complex mathematical computation derived in the above-mentioned previous studies [82]. Using${\mathrm{MMG}}_{p-p },{\mathrm{MMG}}_{\mathrm{RMS}}$, the knee angle, and the levels of NMES parameters, the SVR was trained to predict torque; then, the knee torque was in line with the dynamometer readings. The high correlation and reduced root mean square error only confirmed the efficiency of the developed technique in terms of estimating the knee torque and the accuracy of the laboratory results. Similarly, the variability in the neurological function of SCI patients determined in this study [83] was addressed using artificial intelligence, which was performed in a subsequent study [86]. After delivering electrical impulses to the quadriceps muscle groups of three SCI subjects, the knee torque estimated using a dynamometer was significantly correlated with the ANN-predicted torque. Therefore, the authors highlight the usefulness of ANN for real-time torque estimation.

Thereafter, the methods were further validated in a later study [84]. Using increasing stimulation amplitudes, the peak-to-peak torque was obtained from an average of the 20 highest data points, and the M-wave, H-reflex, and MMG responses were also determined using the peak-to-peak amplitudes. Thus, the combination of M-waves and H-reflexes was proposed for estimation of the plantar flexion torque using Cybex 6000. The study then introduced the M + H parameter, which was strongly correlated with the twitch torque and MMG. Hence, the study was able to determine that MMG was not affected by stimulus artifacts and is thus recommended for estimating the maximal twitch torque. This pioneering work on NMES and mathematical models continues to guide research in the characterization of muscles, particularly small muscles. Using SVD, the transfer function between torque and MMG reported by a previous study [85] showed that the activity of small, thin, and parallel muscle fibers can be mathematically modeled through a combination of DMMG and AMMG. The findings of these studies have supported the use of muscle mechanics, anthropometry, and age difference in terms of characterizing the muscle torque. Machine learning [83, 86] was also determined to provide greater results which support the hypothesis that sophisticated signal processing may provide promising future improvements in terms of MMG-based muscles studies.

### Miscellaneous/clinical monitoring

MMG can reportedly be used in real-time clinical monitoring [88–93]. The authors of a related study [92] were able to detect an MMG response of the target nerve to a specific stimulation current to verify the rate of nerve root decompression, wherein a decrease in the stimulation threshold of ≥ 1 mA was observed in 98% of the test subjects. The affected nerve root maintained a stimulus threshold of 1 mA following decompression but exhibited an increase in the amplitude of the MMG signal response. The technique can thus adequately guide a surgeon in cases in which decompression is deemed uncertain. Furthermore, two other studies [88, 89] verified the validity of KMG and PMG in neuromuscular monitoring. Prior to general anesthesia, the ulnar nerve has received supramaximal train-of-four stimulation for every 12 s at 0–70 mA. Based on the concordance correlation coefficient, the recovery of NMB determined using KMG and PMG agreed with that obtained based on the MMG response. In contrast in [86], the features of KMG appear to exhibit a high TOF ratio in NMB monitoring. Some researchers have compared the KMG data recorded using a Datex M-NMT MechanoSensor with EMG data recorded with a Datex ElectroSensor and found a higher recovery in terms of TOF % with KMG compared with EMG. However, the researchers stated that the 90% recovery rate of KMG makes it less preferable for NMB than EMG. Thus, further validation of these findings in clinical settings is needed [90].

In another study [93], the BB was synchronously stimulated with a programmable function generator providing a 1-ms pulse, and 3D images were acquired through ultrafast acquisition in the transverse plane at each position. The Tc and $1/2\mathrm{ Tr}$ matched the in vitro and in vivo surface MMG responses. In addition, the muscle fiber bundle was retrieved and agreed with the echogenic architecture of the muscle. Further, the researchers argued that the technique can be used to remedy the inability of diffusion tensor imaging in providing the mechanical function of a muscle, allowing for easy recruitment of muscle fibers. Therefore, the authors encourage further verification of the validity of myography-based screening of the functions of other muscles.

Several of the available techniques for respiratory assessment have been determined to involve maximal inspiratory and expiratory mouth pressures. After electrical excitation of the phrenic nerve, the MMG responses from the contracted diaphragm correlated with the respiratory muscle strength and provided higher sensitivity than the conventional method [91]. Altogether, the techniques of MMG, KMG, and PMG through NMES are presented as interesting tools to evaluate muscle behaviors in clinical setting with no physical efforts. Taking advantage of the myographic signals from NMES to monitor specific muscle status, more research is needed to verify the functions of other muscles.

## Conclusion

The studies examined in this review presented a comprehensive overview of approaches used for assessing muscle activities based on MMG through electrical activation protocols. The technique has been used to identify the contractile properties of muscles in specific tasks to denote the changes that take place after electrical activation for recovery and function monitoring. Our findings support the hypothesis that MMG coupled with NMES is suitable for muscle function assessment. However, like EMG, the MMG signal alone is prone to crosstalk contamination from nearby muscles and those beneath the skin. Coupled with electro-muscle stimulation, unlike EMG, the MMG signal showed no interference from electrical signals. Thus, taking advantage of electrical excitation, the examination of the activity of a single muscle from a group of muscles could be a tool to quantify the crosstalk. In this review, it was also shown that a combination of MMG, NMES, and task training provided robust outcomes. Hence, disabled people can be psychologically motivated, thus familiarizing with electric sensation in functional training. Nevertheless, the application of NMES differs depending on one’s susceptibility to intensities of stimulation and the degree of disability when applied for rehabilitation purposes. It will be important to establish if MMG and electrical muscle stimulation can yield a functional rehabilitation program for people with or without complete SCI. Studies have shown that MMG parameters obtained through electrical muscle stimulation protocols have reliable physiological parameters of targeted muscles that correlate with torque and have been also used for fatigue, stiffness, and endurance assessments of skeletal muscles using mathematical computations. Our findings from the statistical analysis of MMG and electromyostimulation coupling suggest the use of artificial intelligence as a potential new approach.

## Data Availability

Not applicable.

## Abbreviations

Delay_TOT_: Delay in force development
½T_r_: Half relaxation time
Ag–PDMS: Silver–polydimethylsiloxane
AMMG: Acceleration mechanomyography
ANN: Artificial neural network
CDS: Contact displacement sensor
DGR: Down-going ramp
*D*_m_: Maximum displacement
DMMG: Displacement mechanomyography
EMG: Electromyography
FRDA: Friedreich ataxia
GLM: Gloss lateral movement
GM: Gastrocnemius
ICC: Intraclass correlation coefficient
IED: Inter-electrode distance
KMG: Kinemyography
LCE: Leg cycling exercise
LDS: Laser displacement sensor
LFF: Low-frequency fatigue
MCS: Muscle contraction sensor
MEMS: Microelectromechanical system
MF: Mean frequency
MFCC: Mel frequency cepstral coefficient
MMG: Mechanomyography
MPF: Mean power frequency
MTU: Muscle–tendon unit
MU: Motor unit
MVC: Maximum voluntary contraction
MVIC: Maximum voluntary isometric contraction
NIRS: Near-infrared spectroscopy
NMES: Neuromuscular electrical stimulation
PAP: Post activation potentiation
pT: Peak torque
RF: Rectus femoris
RMS: Root mean square
SCI: Spinal cord injury
sEMG: Surface electromyography
SOL: Soleus
SVD: Singular value decomposition
SVM: Support vector machine
SVR: Support vector regression
TB: Triceps brachii
TMG: Tensiomyography
UGR: Up-going ramp
VL: Vastus lateralis
VM: Vastus medialis
VMG: Vibromyography
VML: Vastus medialis longus
VMO: Vastus medialis obliquus
